# DNA Sequence Evolution and Rare Homoeologous Conversion in Tetraploid Cotton

**DOI:** 10.1371/journal.pgen.1006012

**Published:** 2016-05-11

**Authors:** Justin T. Page, Zach S. Liechty, Rich H. Alexander, Kimberly Clemons, Amanda M. Hulse-Kemp, Hamid Ashrafi, Allen Van Deynze, David M. Stelly, Joshua A. Udall

**Affiliations:** 1 Biology Department, Brigham Young University, Provo, Utah, United States of America; 2 Plant and Wildlife Science Department, Brigham Young University, Provo, Utah, United States of America; 3 Department of Soil & Crop Sciences, Texas A&M University and Texas A&M AgriLife Research, College Station, Texas, United States of America; 4 Seed Biotechnology Center, University of California-Davis, Davis, California, United States of America; John Innes Centre, UNITED KINGDOM

## Abstract

Allotetraploid cotton species are a vital source of spinnable fiber for textiles. The polyploid nature of the cotton genome raises many evolutionary questions as to the relationships between duplicated genomes. We describe the evolution of the cotton genome (SNPs and structural variants) with the greatly improved resolution of 34 deeply re-sequenced genomes. We also explore the evolution of homoeologous regions in the A_T_- and D_T_-genomes and especially the phenomenon of conversion between genomes. We did not find any compelling evidence for homoeologous conversion between genomes. These findings are very different from other recent reports of frequent conversion events between genomes. We also identified several distinct regions of the genome that have been introgressed between *G*. *hirsutum* and *G*. *barbadense*, which presumably resulted from breeding efforts targeting associated beneficial alleles. Finally, the genotypic data resulting from this study provides access to a wealth of diversity sorely needed in the narrow germplasm of cotton cultivars.

## Author Summary

The polyploid genome of domesticated cotton contains two different copies of most genes because its genome is duplicated. Instead of 13 chromosomes like its wild relatives, domesticated cotton has 26 chromosomes (13 A_T_ chromosomes and 13 D_T_ chromosomes). Differences in the gene copies may hold keys to the genetic improvement of cotton. In fact, it has been thought that the two copies in the cotton genome interact in an unexpected way called gene conversion. In regular diploid genomes, gene conversion occurs when the maternal copy is used to ‘fix’ or ‘overwrite’ the paternal copy (or *vice versa*) during cell division. In cotton, this mechanism of conversion has been used to explain small DNA changes between genomes over evolutionary time. However, we do not see any evidence for conversion events in our new, large sequencing datasets. Our datasets and methods are much more robust than previous reports. Correction of the idea that “extensive homoeologous gene conversion is common in cotton” is important because 1) the cotton genome is used as a model for plant genome research and 2) efforts to induce homoeologous gene conversion in cotton breeding would be unsuccessful. In addition, this report discovers a large set of single-base changes (SNPs) among cotton varieties and makes them available to the research community for public use.

## Introduction

Domesticated cotton has a polyploid genome consisting of an A_T_- and D_T_-genome (the “T” subscript indicates tetraploid nucleus). Approximately 1 mya, a polyploidization event gave rise to six described AD allotetraploid species with genome sizes ~2400 Mbp, mostly native to Central and South America [1–4]. The A_T_-genome (1700 Mbp) is ~2-fold larger than the D_T_-genome (900 Mbp) and there is approximately a 2-fold greater genetic distance between the related diploid *G*. *raimondii* (D_5_) and D_T_ than between the related diploids *G*. *herbaceum* (A_1_) or *G*. *arboreum* (A_2_) and A_T_. There are two major clades among the six tetraploid species, one containing *G*. *hirsutum* (AD_1_) and the other containing *G*. *barbadense* (AD_2_). Both of these species were independently domesticated and produce long spinnable fiber. The remaining tetraploid species (AD_3_ –AD_6_) AD_1_ is the source of the vast majority (~90%) of worldwide cotton production [5]. AD_2_ accounts for another ~5%; its longer fibers are valued for high quality textiles. Attempts to produce stable AD_1_ x AD_2_ hybrids have resulted in fertile and productive F_1_ hybrids, but development of hybrid seed is generally cost-prohibitive. In addition, hybrid breakdown, hybrid sterility, and selective elimination of genes make genomic resources difficult to develop. As such, introgression of genetic material from AD_2_ into AD_1_ (or *vice versa*) is of particular interest.

While introgression between species increases their respective genetic diversity, conversion events between sub-genomes of a polyploid would reduce diversity within a genome. Homoeolog conversion—also called gene conversion, non-reciprocal homoeologous recombination, or homoeologous gene conversion—is a phenomenon in which an allele from one genome of a tetraploid overwrites its homoeolog in the other genome. For example, a D_T_-genome allele overwrites its A_T_-genome homoeolog, resulting in 4 copies of the D_T_-genome allele and 0 of the A_T_-genome allele, instead of 2 of each as would normally be expected. Homoeologous conversion has been identified in various tetraploid groups, including *Brassica* [6,7] and *Gossypium* [8,9]. Homoeologous conversion may be caused by non-reciprocal homoeologous recombination or other sources of double-strand break repair, although the specific mechanisms or causes for such events is still uncertain. It has been hypothesized that homoeologous recombination is a major force in the evolution of desirable traits in allopolyploid crops [10], suggesting that it may be the reason that fiber traits in cotton have been selected on the D_T_-genome. The majority of genetic diversity among allopolyploid cotton species has been attributed to homoeologous conversion [11].

Identification of homoeologous conversion events using short read data from cotton or other allopolyploid genera requires specialized software. We have identified and implemented two different strategies to categorize mapped reads from tetraploid cotton to their genome of origin: PolyCat [12] and PolyDog [13]. Both programs are freely available as part of BamBam [14] at https://sourceforge.net/projects/bambam/ and were used as part of this study. PolyCat uses SNP-tolerant mapping of GSNAP [15] with an index of known homoeo-SNPs (SNPs that differentiate the A- and D-genomes) instead of its traditional use to index SNPs in the human genome sequence. Consequently, the reads are aligned to a single diploid sequence (representing a relative of one of the parent genomes) with minimized mapping bias between genomes. The end result of these strategies are sets of reads that belong to the A_T_- or D_T_-genomes (in addition to reads that do not overlap a homoeo-SNP). Reads separated by genome provide a rich dataset for genome analyses within and between sub-genomes.

The results of these analyses provide insight into genetic diversity, evolution, and specific traits of specific plant species. Previous re-sequencing efforts of other domesticated plant genomes such as corn, tomato, and cotton diploids have investigated mutations, selection, and linkage disequilibrium [16–18]. In this study, we apply Illumina technology to re-sequence and compare the genomes of 34 cotton tetraploids from 6 species at average coverage 23x per accession, whereas previous cotton tetraploid resequencing efforts have used only minimal coverage. We we examine the comparative evolution and genetic diversity of the polyploid cotton species and genomes by mapping reads to the diploid A- and D-genome reference sequences of *G*. *arboreum* [19] and *G*. *raimondii* [20], as well as to the recently published drafts of the cotton tetraploid genomes [21,22]. Mapping to the diploid sequences for this report is tenable because 1) the A_T_- and D_T_-genomes do not have common loci positions and 2) >25% of the draft tetraploid sequences remain formally unanchored to either A_T_- or D_T_-genomes. Much of our study included comparisons between A and D (or A_T_ *vs*. D_T_), and the comparisons are only possible in regions present in *both* A and D genomes, making the draft tetraploid sequences less informative. To improve results based on diploid sequences, we account for the differences between the respective diploid and tetraploid genomes by adjusting the diploid reference sequences to the genotypes observed in the tetraploid species.

## Results

### Mapping and Categorization

**Fig 1:**
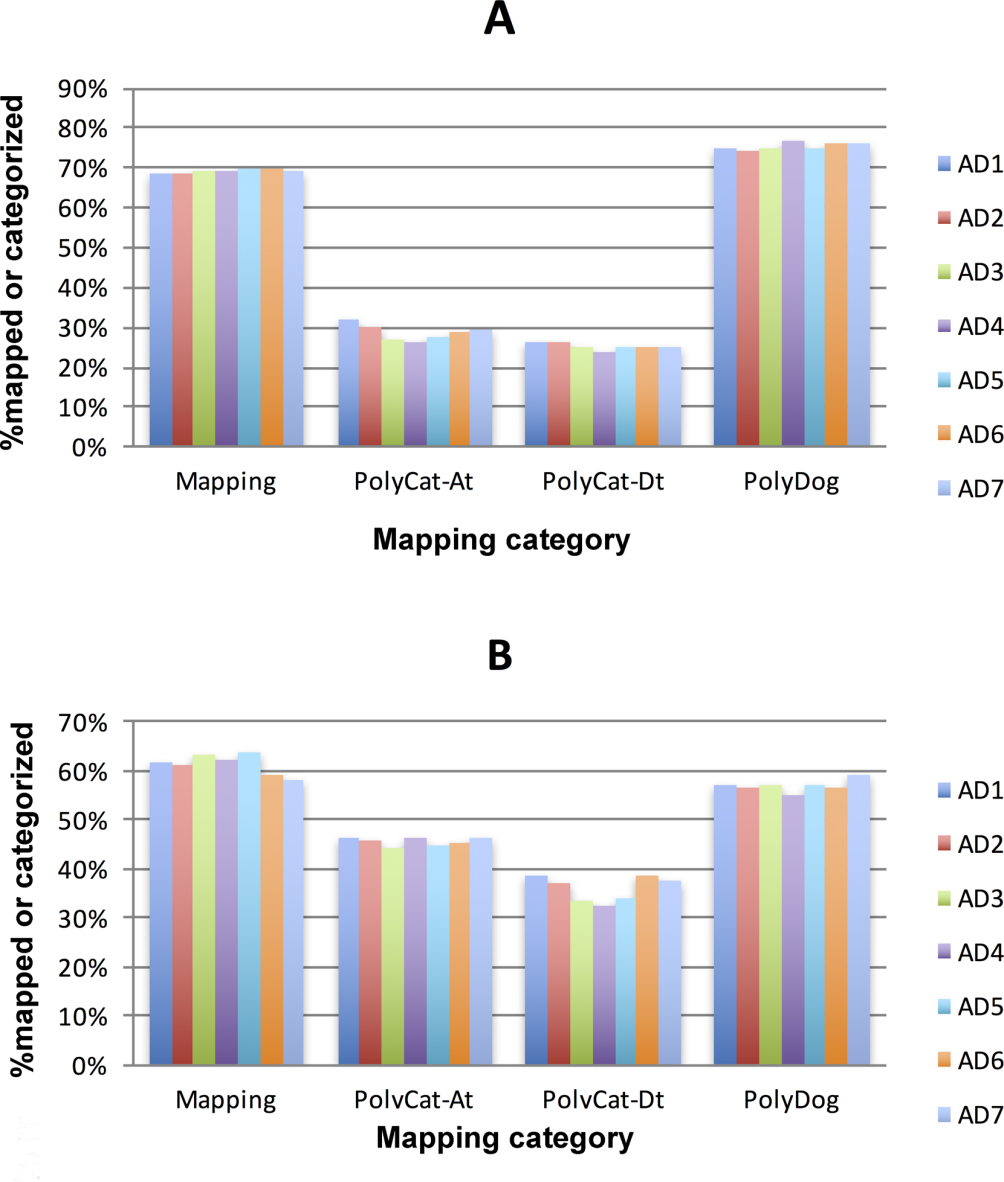
Effectiveness of mapping with GSNAP and categorization with PolyCat and PolyDog. PolyDog has higher categorization rates because it allows for genome-wide categorization, instead of relying on known homoeo-SNPs in regions present in both genomes. The percentage of trimmed reads successfully mapped from each species (AD_1_-AD_7_) to the A_2_ reference (A) and the D_5_ reference (B) is shown. For each reference, the percentage of mapped reads from each species (AD_1_-AD_7_) categorized to the A_T_-genome by PolyCat, D_T_-genome by PolyCat, or to the genome of the reference sequence by PolyDog is also shown.

PolyDog read mapping and categorization uses a complete representation of the tetraploid genome by mapping each set of reads to both diploid reference genomes of A_2_ and D_5_. Approximately 60% of reads from tetraploids mapped to unique loci on the D_5_ reference, while 70% mapped to unique loci on the A_2_ reference (Fig 1). The larger mapping percentage for the A_2_ reference is likely because the A_T_-genome is larger than the D_T_-genome, so more reads drawn randomly from the tetraploid should be A-like than D-like. The difference is only 10% because much of the extra A-genome sequence is either repetitive (preventing unique mapping by short reads) or simply absent from the reference sequence. More reads were categorized by both PolyCat and PolyDog to the A_T_-genome than to the D_T_-genome. This is likely due to 1) the larger size of the A-genome and 2) the greater genetic distance between D_5_ and D_T_, which slightly decreases the effectiveness and accuracy of read categorization. When using the A_2_ reference instead of the D_5_ reference, the frequency of categorization was lower because less homoeo-SNPs have been defined in the A_2_ reference SNP index. In addition, a greater fraction of the A_2_ reference is non-homoeologous sequence, resulting in more reads that map to the reference but will not be able to be categorized because they only map to A-genome unique sequence. More reads overall were categorized by PolyDog than by PolyCat because PolyDog is able to categorize these reads mapped to non-homoeologous regions [18]. Categorization error rates were measured by mapping diploid reads to each diploid genome (S1 Table). The end result of read mapping and categorization was a read alignment (BAM) file for each genome (A_T_ and D_T_) in each tetraploid accession.

We also mapped reads to the tetraploid TM-1 reference sequence [20]. The numbers of mapped and categorized reads were less than those obtained with PolyDog using the diploid reference sequences. In addition, a significant percentage of the tetraploid sequence was unanchored to either an A_T_- or D_T_-genome. Unanchored scaffolds could be due to either partial assembly or mis-assembly. Thus, further analyses did not use the tetraploid sequence as a genome reference (S1 Table). Eventually, additional improvement of the reference tetraploid sequences may provide better rates of read mapping than PolyDog, but PolyDog is currently the most thorough method of mapping polyploid reads in cotton.

### Single Nucleotide Polymorphisms

We analyzed evolutionary relationships by examining SNPs among the PolyDog-categorized reads. Within read alignments, we identified SNPs between genomes (termed “homoeo-SNPs”) and between accessions (“allele-SNP”). Homoeo-SNPs were first identified between the diploids A_2_ and D_5_ and then between the PolyCat-categorized A_T_- and D_T_-genomes of AD_1_, AD_2_, AD_3_, AD_4_, and AD_5_ (*i*.*e*. between sub-genomes). Between 19.2 and 28.5 million homoeo-SNPs (35.6 million total unique loci) were found when using the D_5_ reference (Table 1). There were 11.2 million homoeo-SNPs positioned on the D_5_ reference sequence that were shared within all tetraploid species (S1 Fig). Of these homoeo-SNPs, 9.4 million homoeo-SNPs were shared within all tetraploid species *and* they were also found between the diploid genomes. About 12–15% of homoeo-SNPs were in annotated genes. There were 1,358 genes with no homoeo-SNPs identified in the tetraploid sequences aligned to the D_5_ reference sequences. These SNPs are available on CottonGen as D13.snp4.x, where x = 0 for homoeo-SNPs found in the diploids, x = 1 for AD_1_, x = 2 for AD_2_, etc.

**Table 1:** Homoeo-SNPs identified between the A- and D-genomes of the diploids and A_T_- and D_T_-genomes of the tetraploids.

	A_2_-reference			D_5_-reference		
	Genomic	Genic		Genomic	Genic	
Diploids	15,618,185	2,090,126	13.4%	28,540,537	3,009,100	10.5%
AD_1_	18,253,297	2,303,433	12.6%	24,908,821	3,069,346	12.3%
AD_2_	17,286,282	2,224,161	12.9%	24,776,502	3,003,401	12.1%
AD_3_	12,574,385	2,044,681	16.3%	19,235,460	2,742,627	14.3%
AD_4_	12,442,214	1,973,277	15.9%	19,274,313	2,656,550	13.8%
AD_5_	12,914,212	2,017,762	15.6%	19,809,248	2,719,911	13.7%

We identified allele-SNPs within sub-genomes, between accessions of each species, using PolyDog-categorized reads. After filtering SNPs (<10% minor allele frequency), there were 15,864,224 and 10,437,663 allele-SNPs in the A_T_- and D_T_-genomes, respectively. In both AD_1_ and AD_2_, the number of A_T_-genome allele-SNPs was about 1.5x the number of D_T_-genome allele-SNPs (Table 2). Although breeding strategies typically ignore the genome size difference between A_T_ and D_T_, the average diversity (allele-SNPs per bp) in the D_T_-genome was nearly 2x greater than the average diversity in the A_T_-genome after normalizing by genome size. Most of this SNP diversity was intergenic. Within gene annotations, less allele-SNPs were found in the A_T_-genome (947,157 allele-SNPs) than in the D_T_-genome (1,638,565 allele-SNPs; S2 Fig). There were 1,173 genes that had 0 allele-SNPs in the A_T_-genome while their respective homoeologs had 5 or more allele-SNPs in the D_T_-genome. There were 1,835 genes that had 0 allele-SNPs in the D_T_-genome while their respective homoeologs had 5 or more allele-SNPs in the A_T_-genome (S3 Fig).

**Table 2:** SNPs and average diversity (# pairwise differences / # polymorphic sites covered by both individuals) among sub-groups of diploids and tetraploids (AD_1_clade = all accessions on the AD_1_ branch; AD_1_dom = domesticated *G*. *hirsutum* accessions).

	At		Dt	
Group	SNPs	Diversity	SNPs	Diversity
all	27,447,974	0.165%	21,476,013	0.285%
AD	15,864,224	0.132%	10,437,663	0.179%
AD_1_clade	9,555,028	0.060%	6,574,982	0.099%
AD_1_dom	7,875,126	0.048%	5,610,018	0.092%
AD2	9,489,947	0.048%	6,376,241	0.085%

### Copy Number Variants

**Fig 2:**
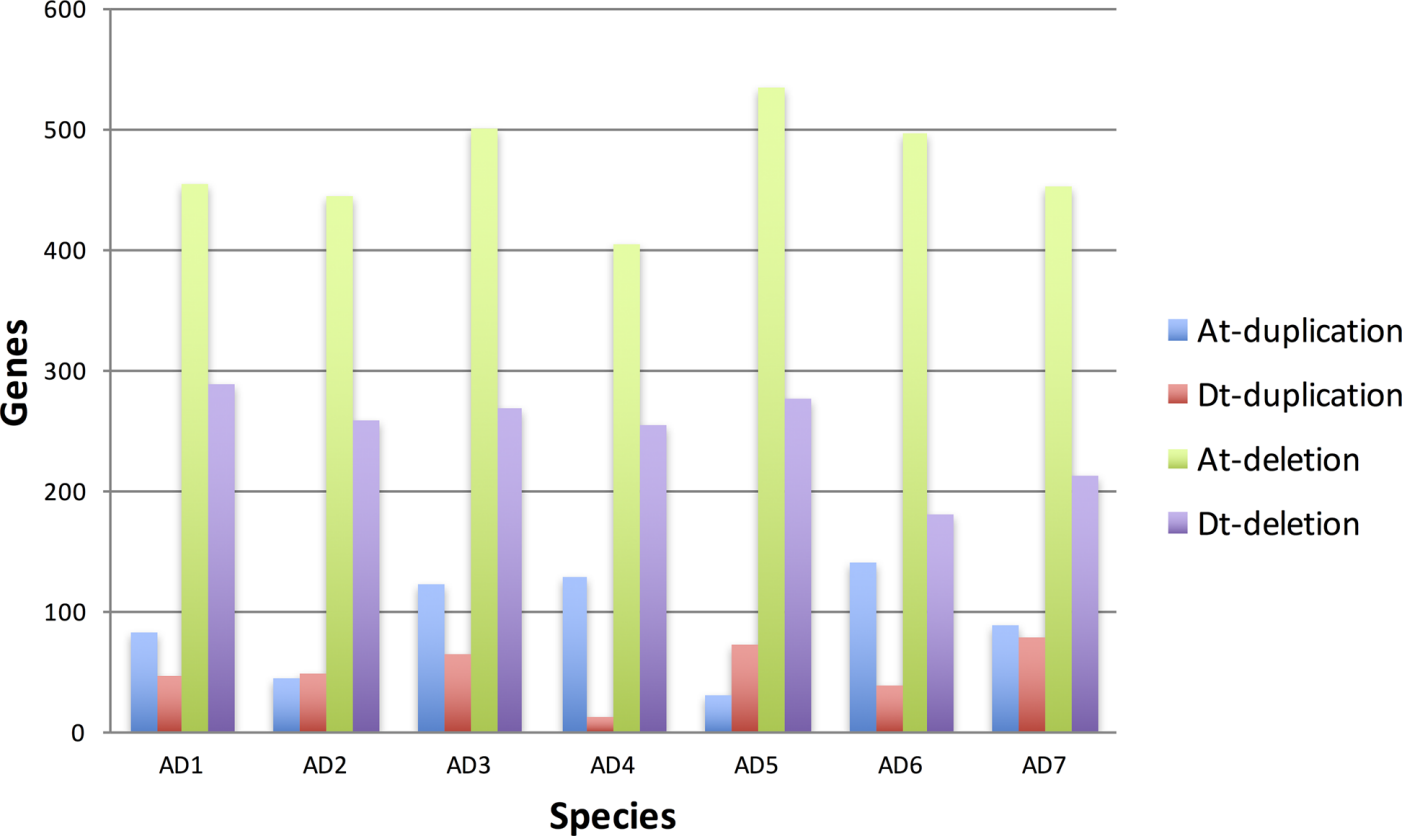
Copy number variants were generally more common in the larger AT-genome than in the smaller DT-genome, and deletions were more common than duplications, in accordance with the idea of reciprocal gene loss. Duplications and deletions were identified in each genome of each species, relative to the extant diploid relative. CNVkit detected CNVs and determined their sizes using a log base 2 threshold of 1.0 for duplications and -1.0 for deletions. Blue indicates duplications in A_T_-genomes compared to A_2_ individuals. CNVkit identified duplications and deletions, with a minimum threshold of 2-fold difference.

Copy number variants (CNVs) indicate regions of historic duplication and/or deletion, and there are various strategies used to identify them [23,24]. CNVs were detected in the ‘continuous-coverage’ of PolyDog-categorized BAM alignment files by CNVKit [24]. Deletions in the A_T_-genome were the longest and most common type of copy number variant, with ~69 blocks and 19 Mbp per accession (Fig 2; S2 Table). Deletions in the D_T_-genome were much less frequent, with ~31 blocks and less than 5 Mbp per accession. Duplications were considerably less frequent than deletions, with less than 10 blocks and 1 Mbp per accession. In the D_T_ genome, a similar number of duplications were found in AD_1_ and AD_2_, but A_T_-genome duplications were more common in AD_1_ than in AD_2_. No pattern in frequency of duplications or deletions appeared to distinguish wild and domesticated lines. In comparisons between species, AD_4_ had few duplications and deletions, and had a particularly low number of D_T_-genome duplications. Certain combinations of overlapping CNVs were also used to detect homoeologous conversion events (see below).

Deletions were much more conserved than duplications, although this is likely related to the larger number of deletions detected because shared deletions more likely to occur by chance (S3 Table). The limited number of gene deletions suggest that the sub-genomes within the polyploid have not diploidized, and that there very small differences in the amount of genome fractionation (*i*.*e*. gene loss) between sub-genomes. Consequently, we use the term ‘sub-genome’ sparingly (when needed for reader clarity) because one genome is not nested inside another genome, and evidence that the A_T_- or D_T_-genomes (*i*.*e*. sub-genomes) are less than a complete genome is very limited (S3 Table). Duplications in the A_T_-genome were more conserved than duplications in the D_T_-genome, but duplications differed greatly from accession to accession, even among the closely related AD_1_ cultivars. Generally, conservation rates of CNVs were higher in cultivars than in wild accessions and could have been the result of a recent shared ancestry.

### Homoeologous Conversion

A new homoeologous conversion event would result in a long series of consecutive conversion-SNPs and overlapping duplications/deletions between homoeologs. Given the 5–10 million-year history of nuclear co-residency, the conversion events would be somewhat fractionated by historical recombination or by mutation accumulation. Two approaches were used to investigate genome conversion events in cotton: SNPs and overlapping CNVs. The SNP-based method would detect older homoeologous conversion events that have subsequently been obfuscated over time. The CNV method would detect recent conversion events. Events between the two temporal extremes should be faintly detected by both methods, though the date of polyploidization provides a hard time limit to how ‘ancient’ conversion events may actually be.

In the first approach, gene conversion was detected by a parsimony-based method of SNPs, similar to that employed by other studies [8,9,11,21,25]. Reads were categorized to the A_T_- and D_T_-genomes with PolyCat, in order to allow intergenomic comparison at a nucleotide level. Genotypes were called using InterSnp with a minimum allele coverage of 5 reads. Polymorphic loci were selected where 75% of individuals had an alternate allele. These were tested for a genotype pattern indicative of homoeologous conversion in *G*. *hirsutum* and *G*. *barbadense* by comparing the tetraploid genotypes to the diploids (as a proxy ancestor genotype). However, the diploids A_2_ and D_5_ do not precisely represent the true progenitors of the A_T_- and D_T_-genomes [5]. Mutations that have occurred in the extant diploid after their divergence from the progenitors of the polyploid will result in false positive events of simple conversion detection because both tetraploid genomes will match the diploid that did not have the mutation. For example, the equivalence of A_2_ = A_T_ = D_T_ ≠ D_5_ could be due to a mutation in the D_5_ lineage (a D_5_ autapomorphism), rather than to a homoeologous conversion.

To correct for diploid autapomorphies, we use the AD_4_ as an outgroup for AD_1_ and AD_2_ intra-genome comparisons [4,26]. If a putative homoeologous conversion was detected in AD_4_ as well as in AD_1_ and/or AD_2_, then it was due to 1) a conversion event immediately after (or coincidental) with polyploidization or to 2) an autapomorphic mutation unique to one of the diploid lines [8,9,12]. Using the D_5_ reference sequence, 1,322,948 putative A-dominant events were found in AD_1_ and could be compared to AD_4_. Of those, only 52,680 (4.0%) were putative homoeologous conversion events after compared to the AD_4_ sequence. The remaining 1,270,268 were false positives (autapomorphies in the D_5_ diploid) or possibly occurred immediately after polyploidization. Similar numbers were observed for AD_1_ and AD_2_ (S4 Table). A greater percentage of D-dominant conversion events were found: 65,276 (6.7%) out of 979,045. We repeated this analysis using the A_2_ genome reference. This change of reference sequence resulted in detection of a similar number of events, but more A-dominant than D-dominant conversions. This suggests that the choice of reference sequence may be a source of false positive events. Similar ratios of ‘true’ (AD_4_-considered) and false (AD_4_-ignored) conversion events were observed in AD_1_ and AD_2_, and a little less than half of the likely homoeologous conversion loci were shared by AD_1_ and AD_2_ suggesting events prior to the division between the AD_1_ and AD_2_ clades.

The number of conversion events can also be examined by considering consecutive, putative conversion-SNPs because not every pair of ‘ancient’ conversion-SNPs from a single event would not have been interrupted by recombination or by mutations. Very few consecutive loci in the genome supported homoeologous conversion and most were two-consecutive SNPs and not a larger series of consecutive SNP loci (Table 3). As with the conversion-SNPs discussed in the previous analysis, many more regions of consecutive homoeologous conversion SNPs were detected as dominant for the same genome as the reference. When the A_2_-reference sequence was used, fewer consecutive SNPs representing fewer regions were found, but they overlapped more genes. Thus, we found that nearly all of the SNP-based evidence for genome conversion to be indistinguishable from coincidental mutational noise within AD_4_ and other polyploid genomes, and from error inherent to our SNP-detection methods (*e*.*g*. choice of genome reference *etc*.).

**Table 3:** Regions including 2 or more consecutive ancient gene conversion SNPs provided little SNP-based evidence for sequence conversion between genomes.

		A_2_-reference		D_5_-reference	
Type	Number	AD_1_	AD_2_	AD_1_	AD_2_
A_T_-dominant	SNPs	3,145	2,662	2,491	2,636
	Regions	818	699	640	697
	Total Length (Kbp)	1,636	1,327	413	499
	Genes	144	143	8	6
D_T_-dominant	SNPs	401	183	10,769	8,383
	Regions	100	45	2,661	2,097
	Total Length (Kbp)	747	209	3,766	2,793
	Genes	29	14	60	50

A second approach was used to investigate conversion events across regions much larger than the size of a sequence read. In this case, read categorization should mis-categorize reads within converted regions resulting in a duplication of one loci (*i*.*e*. ~2x coverage) and a corresponding deletion (*i*.*e*. no coverage) at its homoeologous locus. In other words, overlapping CNVs (duplications and deletions) can be detected between bam files of A_T_- and D_T_-genome categorized reads. Very few putative homoeologous conversions of this type were detected (Table 4). As mentioned above, more deletions than duplications were found in all of the genomes analyzed and rarely did a deletion in one genome entirely ‘overlap’ a duplication at its homoeologous locus. One large possible conversion event was detected on Chromosome 12, containing nearly all of the genes that are located in regions with evidence of conversion (full or partially converted, S5 Table). This event was also detected in several accessions; however, various additional facts suggest that it was not a true conversion event (although it may have been a true duplication and true deletion): 1) the accessions exhibiting this possible conversion are not monophyletic. They include some accessions of AD_1_ and AD_2_, but not the other members of those species. 2) The duplication associated with this possible conversion event is ubiquitous among tetraploid lines, while the deletion associated with the possible conversion occurred in only a subset of the individuals with the duplication. 3) The duplication/deletion events (deletion events in particular) do not have the same start and stop sites. For these reasons, we suggest that this possible conversion event—the only major event suggested by our sequencing data—was likely not a true conversion event because a complex series of introgression and selection that would be needed to occur to find it in two separate species of cotton. Ascribing the overlap of real duplications and of real deletions to homoeologous conversion event(s) invokes a complicated interpretation to data that may be only coincidental detection of CNVs.

**Table 4:** The number of genes impacted by putative large gene conversion events based on copy number variants (by accession).

Accessions	# Genes
AD_3_	15
AD_5_	16
AD_7_	17
Deltapine-340	15
Fibermax-832	17
PI-265165	15
PI-361153	15
PI-528243	15
AS-828	15
PI-528325	16
M-240	15
Phytogen-76	15
SureGrow-747	15
TX-0231	15
Acala Maxxa	2

### Phylogenetic Relationships

There are six described tetraploid speices of cotton [27]. While AD_1_ and AD_2_ have been domesticated, the remaining tetraploid species (AD_3_ –AD_6_) have not been domesticated because they do not produce spinnable fiber. Another unnamed island endemic of the Northern Line Islands is under consideration as a seventh tetraploid species (Wendel, personal communication; AD_7_). *G*. *ekmanianum* (AD_6_) belongs to the AD_1_ clade and has only recently been described as a distinct species separate from AD_1_ [3]. *G*. *darwinii* (AD_5_) belongs to the AD_2_ clade. *G*. *mustelinum* (AD_4_) diverged from the other tetraploids prior to the divergence between the AD_1_ and AD_2_ clades, making it a useful outgroup for analyses of the cotton tetraploids. The position of *G*. *tomentosum* (AD_3_) from Hawaii is either part of the AD_1_ clade or an outgroup to the split between AD_1_ and AD_2_.

**Fig 3:**
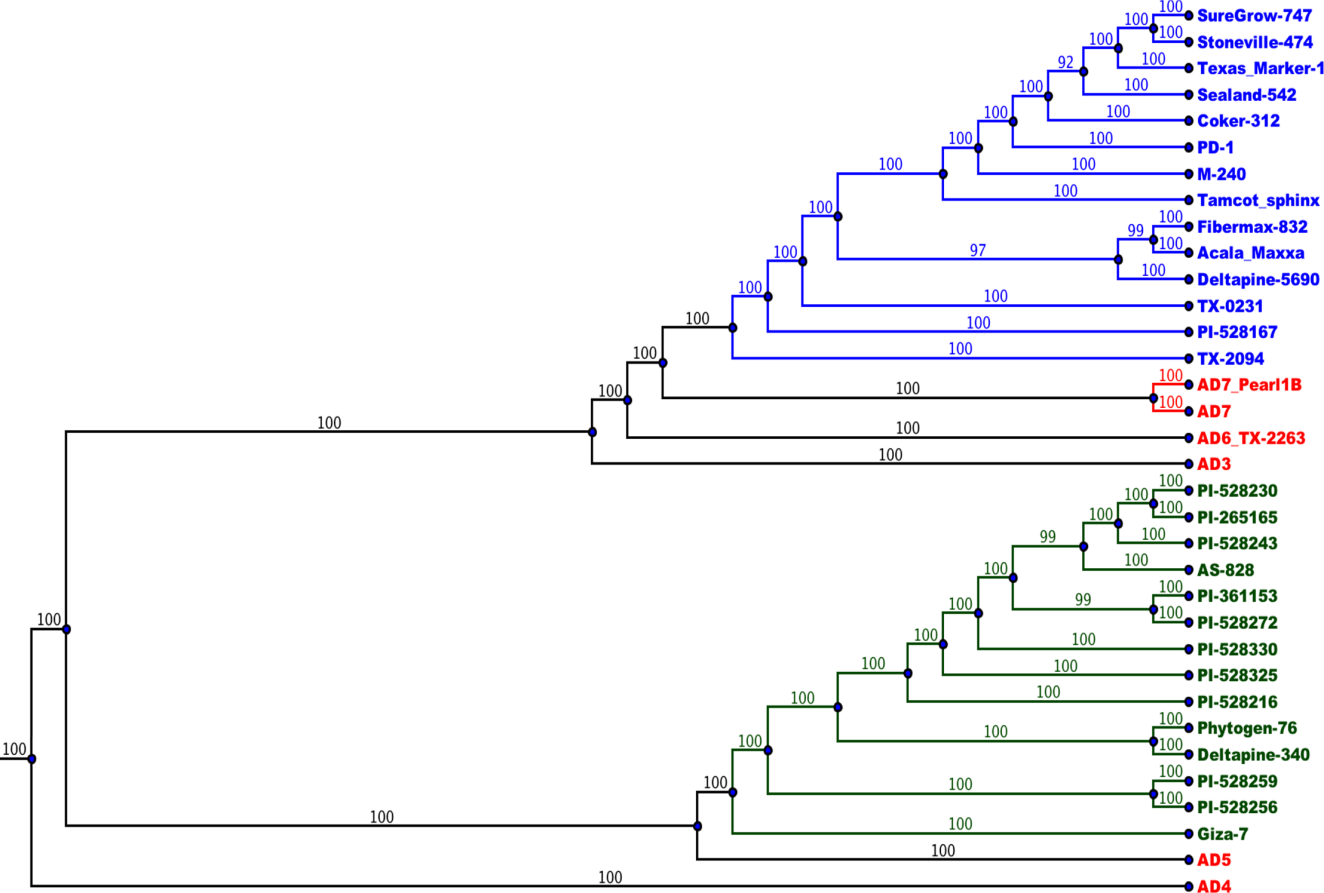
Evolutionary relationships between accessions based on the A_T_-genome. Most accessions are located according to expectation from previous studies and in agreement between the A_T_- and D_T_-genome based trees. Consensus bootstrap neighbor-joining trees were constructed (by PHYLIP) based on distance matrices representing SNPs between each pair of accessions. The root representing the point of connection to the diploid relatives. Individuals from AD_1_ are colored blue, AD_2_ colored green, and other tetraploid species colored red. Branch numbers indicate percent bootstrap support for that split. The A_T-_ and D_T_-genome trees largely agreed in regard to the topology of the AD_2_ clade, with the exception of the positioning of a sub-clade containing the 3 cultivars: Deltapine-340, Giza-7, and Phytogen-76. The AD_1_ clade was similarly constructed in the A_T_- and D_T_-genome phylogenies, although the cultivars are so closely related to one another that their precise arrangements varied between trees.

**Fig 4:**
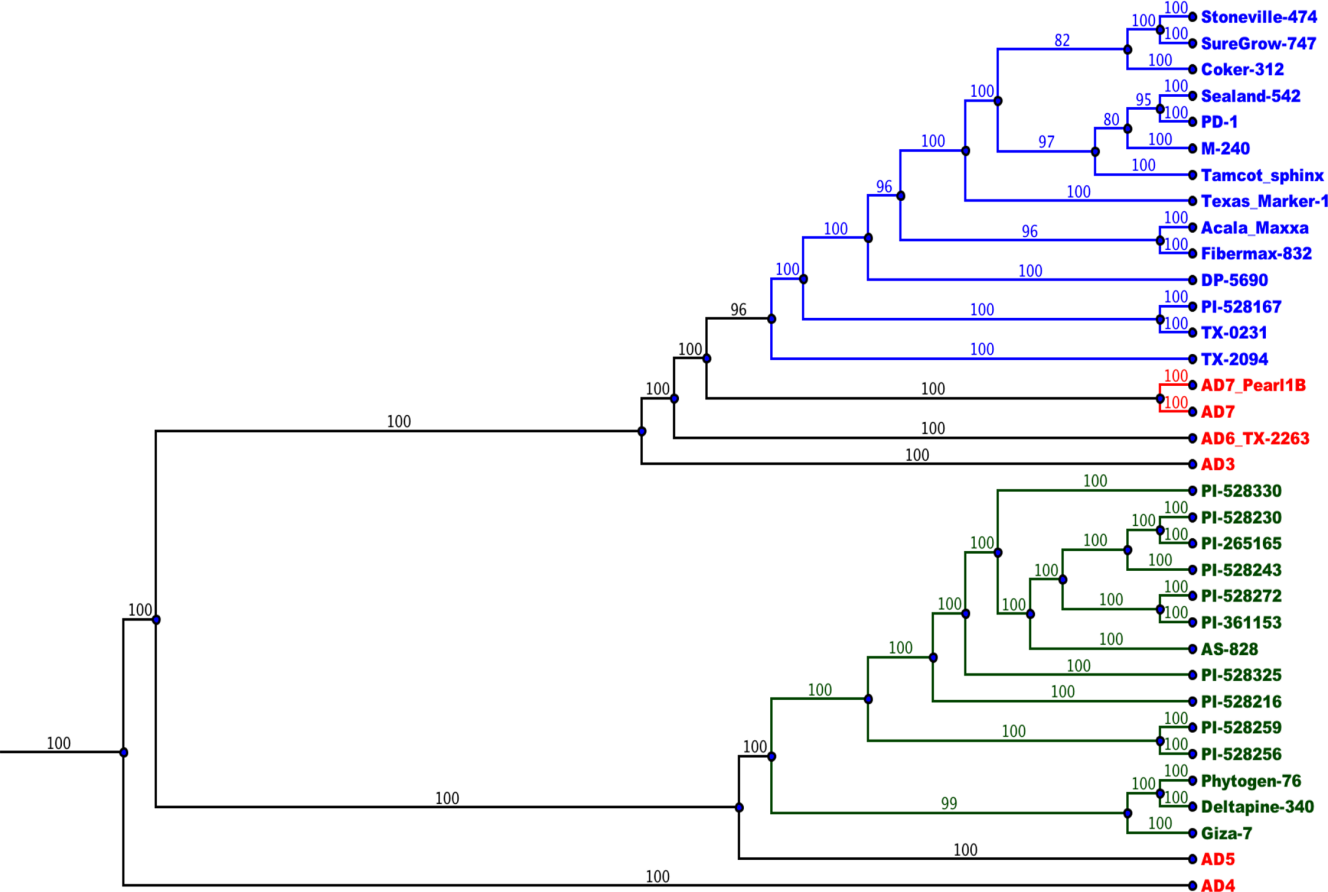
Evolutionary relationships between accessions based on the D_T_-genome. Most accessions are located according to expectation from previous studies and in agreement between the A_T_- and D_T_-genome based trees. Consensus bootstrap neighbor-joining trees were constructed (by PHYLIP) based on distance matrices representing SNPs between each pair of accessions. The root representing the point of connection to the diploid relatives. Individuals from AD_1_ are colored blue, AD_2_ colored green, and other tetraploid species colored red. Branch numbers indicate percent bootstrap support for that split. Outside of the AD_1_ cultivars, the AD_1_ wild accessions (TX-2094 and TX-0231) were closest to the cultivar clade.

The A_T_- and D_T_-genome SNP phylogenies positioned species consistent with previous observations [5,27]. The A-genome donor to the tetraploid lines was similar to extant, diploid *G*. *herbaceum* (A_1_) and *G*. *arboreum* (A_2_), while the closest extant diploid relative of the D-genome donor is likely *G*. *raimondii* (D_5_) [8]. The large number of SNPs between the A- and D-genomes (between diploid and within tetraploid genomes) result in separate monophyletic branches. Thus, separate phylogenetic analyses were performed for the A_T_-and D_T_-genomes. The tetraploids primarily split into two clades, one containing AD_1_ and the other containing AD_2_. AD_4_ is basal to this split. AD_5_ is closely related to AD_2_, while AD_6_ and AD_7_ are close to AD_1_. AD_3_ is in the AD_1_ clade, but diverged shortly after the AD_1_ vs AD_2_ split, making it a more distant relative of AD_1_ than are AD_6_ and AD_7_ (Figs 3 and 4). In separate consensus bootstrap trees for the nuclear genomes, nearly all splits have 99–100% bootstrap support and only 2 splits (both within the AD_1_ cultivars) have less than 90% support (80% and 82%). The cultivated varieties in AD_1_ clustered together with wild AD_1_ accessions nearby (Fig 3), and the same pattern was observed with AD_2_ cultivars and wild accessions (Fig 4). Notably, PI-528167 (although previously classified as an accession of AD_2_) clustered with the wild AD_1_ accessions. The two AD_7_ accessions formed a clade external to the wild AD_1_, and AD_6_ was external to AD_7_.

### Interspecies Introgression

**Fig 5:**
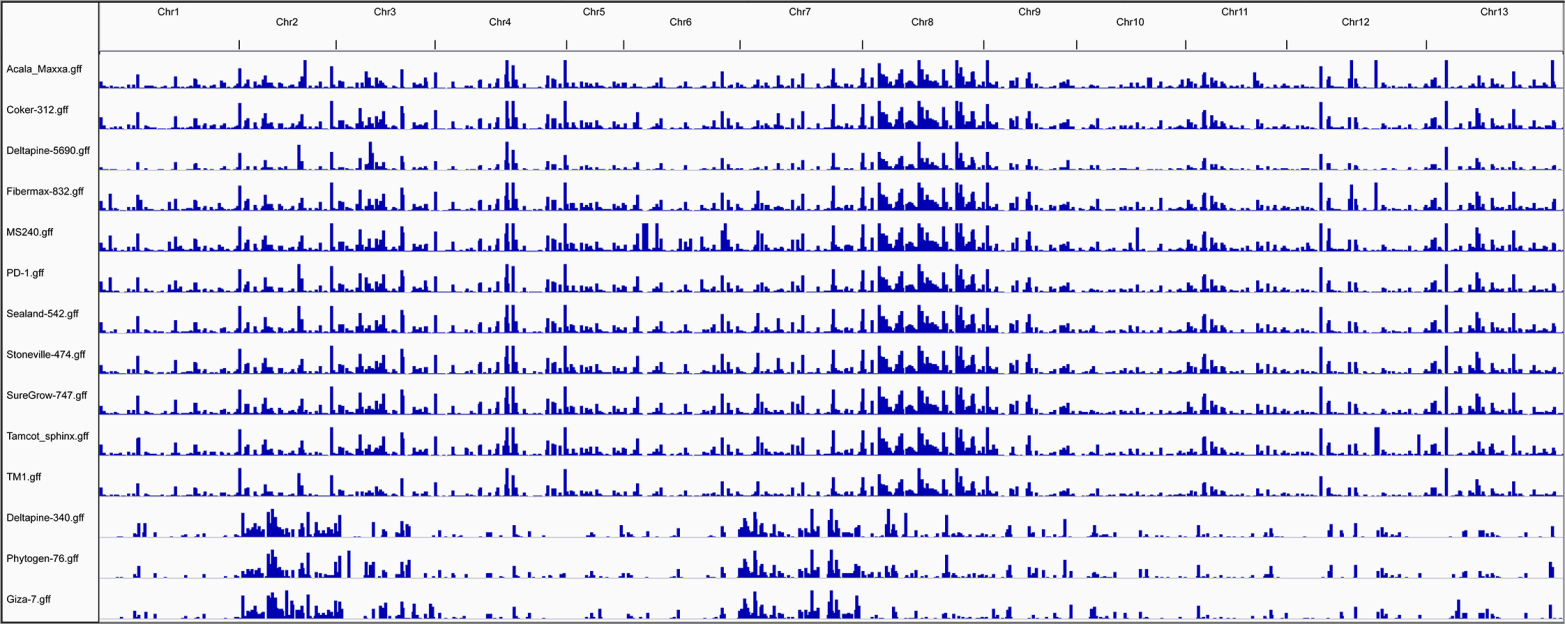
The amount of introgression between *G*. *hirsutum* (AD_1_) and *G*. *barbadense* (AD_2_) varies across the genome A_T_-genome. Wild accessions exhibit a distinct and noisier pattern than cultivars. Wild accessions exhibit a distinct and noisier pattern than cultivars. Regions of introgression are indicated by blue regions of introgression from the ‘other’ cotton species.

**Fig 6:**
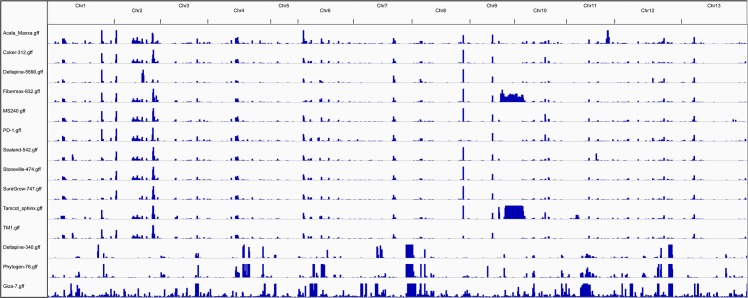
The amount of introgression between *G*. *hirsutum* (AD_1_) and *G*. *barbadense* (AD_2_) varies across the D_T_-genome. Wild accessions exhibit a distinct and noisier pattern than cultivars. Regions of introgression are indicated by blue regions of introgression from the ‘other’ cotton species.

We identified regions of introgression of AD_2_ alleles into AD_1_ cultivars by identifying SNPs between the PolyDog-categorized wild AD_1_ lines (TX-0231, TX-2094, and PI-528167) and AD_2_ lines (excluding PI-528167 because it is not actually AD_2_). The wild AD_1_ lines were used to represent AD_1_ to avoid circularity in SNP examinations of introgression since wild accessions should have negligible amounts of introgression. Consequently, these SNPs provided a method to distinguish alleles that were truly introgressed instead of historical alleles that were ‘unimproved’ in one or more cultivars. Reads from AD_1_ cultivars with bases matching the wild AD_1_ consensus allele were assigned to the “AD_1_-like” category. AD_1_ reads from cultivars matching the consensus AD_2_ nucleotide indicated a locus of putative introgression. There were 3,558,401 and 1,913,744 diagnostic SNPs between the AD_1_ wild lines and the AD_2_ cultivars on the A_T_- and D_T_-genomes, respectively. Using a novel application of PolyCat where these SNPs of introgression were used as a ‘categorizing’ index (as opposed to the standard use of PolyCat that uses homoeo-SNPs as the index), reads from each AD_1_ cultivar were categorized as either wild AD_1_-like or AD_2_-like. Regions with at least 10x coverage of AD_2_-like reads were identified with Eflen (part of BamBam)[14]. Genes in these introgressed regions were identified with BEDTools [28]. On average, each AD_1_ accession had 6.8 Mbp (containing 1,605 genes) of introgression on the A_T_-genome (Fig 5) and 3.8 Mbp (containing 1,934 genes) of introgression on the D_T_-genome (Table 5; Fig 6).

**Table 5:** Evidence for introgression of AD_2_ material into AD_1_ and *vice versa*. Introgression occurred in both directions and in both genomes.

		A_T_ Introgression		D_T_ Introgression	
	Accession	Length	Genes	Length	Genes
**AD**_**2**_ **into AD**_**1**_	Coker-312	6,352,569	1,608	3,495,471	1,887
	Deltapine-5690	2,508,579	640	1,137,344	651
	Fibermax-832	6,650,745	1,809	5,314,686	2,433
	Acala Maxxa	8,558,032	1,998	4,596,128	2,313
	M-240	8,427,778	1,770	3,757,081	1,878
	PD-1	6,556,943	1,564	3,325,321	1,920
	Sealand-542	7,123,054	1,670	3,858,004	1,985
	SureGrow-747	7,656,250	1,604	3,630,665	1,890
	Stoneville-474	6,970,667	1,661	3,704,376	1,936
	Tamcot sphinx	6,606,198	1,750	8,510,980	2,440
	Texas Marker-1	6,817,357	1,605	3,803,304	1,945
	**Average**	**6,748,016**	**1,607**	**4,103,033**	**1,934**
**AD**_**1**_ **into AD**_**2**_	Deltapine-340	21,707,123	2,146	5,878,386	1,938
	Phytogen-76	18,255,558	1,819	4,879,839	1,555
	Giza-7	15,326,627	1,228	4,265,336	1,543
	**Average**	**18,429,769**	**1,731**	**5,007,854**	**1,679**

We performed a similar analysis to look for regions of introgression of AD_1_ alleles into AD_2_ cultivars. Between the AD_1_ cultivars and the wild AD_2_ lines (all AD_2_ except Deltapine-340, Phytogen-76, and Giza-7), we identified 5,217,270 and 2,803,879 diagnostic SNPs on the A_T_- and D_T_-genomes, respectively. As above, only wild AD_2_ lines were used to define “AD_2_-like”, so as to avoid circularity. We then used PolyCat to categorize reads from Deltapine-340, Phytogen-76, and Giza-7 as AD_1_-like or AD_2_-like. On average, each AD_2_ cultivar had 18.4 Mbp (containing 1,731 genes) of introgression on the A_T_-genome and 5.0 Mbp (containing 1,679 genes) of introgression on the D_T_-genome. Interestingly, Giza-7—an obsolete cultivar from the 18^th^ century—had far fewer genes with evidence of introgression than the other cultivars. There was a large difference in the amount of DNA and the number of introgressed genes on the A_T_-genome, suggesting that breeding efforts have not equally focused on both genomes. In addition, the A_T_-genome has more noise (false positive introgression at isolated loci) in the introgression signal than the D_T_-genome, suggesting that one or more of our ‘wild’ AD_2_ accessions had some degree of introgression into their A_T_-genomes.

The PolyCat analysis of introgression easily and robustly identifies areas of putative introgression, but it does not have a formal statistical test or quantitation of introgression. To validate PolyCat’s results, we also tested for introgression into AD_1_ and AD_2_ cultivars (as opposed to wild lines) according to the Patterson D-statistic, which uses three-population trees to measure admixture between genomes as a whole [29]. We performed the test for introgression of Phytogen-76 (AD_2_ cultivar) into 4 AD_1_ cultivars (Maxxa, TM-1, Coker-312, and Tamcot-sphinx) against TX-2094 (wild AD_1_) and of Maxxa (AD_1_ cultivar) into 2 AD_2_ cultivars (DeltaPine-340 and Phytogen-76) against PI-528243 (wild AD_2_). We found strong evidence of cross-species introgression into each cultivar (S6 Table). Further, we again calculated the D-statistic, but only for those PolyCat-predicted regions of introgression. If introgressed regions were correctly identified by PolyCat above, then the D-statistic for those regions alone will be higher than when the D-statistic is calculated for the entire genome. Within the identified regions of introgression, the D-statistic was very high (average 0.90) in each line, validating the PolyCat approach to identify regions of introgression.

## Discussion

### Homoeologous Conversion

In diploid organisms, gene conversion is considered a by-product of recombination where one allele is reconstructed using the second allele as a template [39]. In polyploids, a conversion event that uses homoeologous loci as a template can also result in conversion between ‘sub-genomes’ [8,9,11,21,25]. To distinguish between the traditional definitions of genetic conversion, we refer to the sequence-based events found between genomes (a.k.a. sub-genomes) sharing a nucleus as homoeologous conversions. Homoeologous conversion events were likely caused by historical non-reciprocal homoeologous recombination and it results in a region of a chromosome that is converted to the genotype of its homoeolog. Assuming this region was larger than the size of an sequence read (100 bp reads were used in this study), reads originating in the converted area would be incorrectly categorized as belonging to the homoeologous genome. For example, if the D_T_-genome overwrites a section of the A_T_-genome chromosome, then reads from that region were categorized as D_T_-genome, even though they originated from an A_T_-genome chromosome.

Different methods can be used to search for two different types of homoeologous conversion: small, interspersed regions of SNP patterns (SNP method), and large blocks of homoeologous conversion (CNV method). In the SNP method, a consecutive pattern of shared nucleotides between diploid and tetraploid genotypes along the chromosome suggests homoeologous conversion. The SNP method is discreetly limited by read length, though we required for consecutive SNP occurrences (independent of read length) for homoeologous conversion to be considered. The vast majority of such pattern occurrences—both in our analysis and in that done by Guo et al. [11]—were positioned before the divergence of AD_4_ from the other polyploid species. A pre-AD_4_ homoeologous conversion is indistinguishable (based on extant genotype pattern) from an autapomorphic mutation occurring in one of the diploids. However, the length of time between the polyploidization event and AD_4_ divergence (0 to 0.5 million years) was much shorter than the length of time where such an autapomorphy could occur in one of the diploids (1 to 2 million years). It is therefore likely that the majority of these putative homoeologous conversion events were actually autapomorphic mutations in the diploids.

Examining putative homoeologous conversion events via SNP patterns, we observed 5% (or less) likely homoeologous conversions (as opposed to likely autapomorphic mutations). This value is consistent with EST work predating the use of the reference sequences, which also suggested the possibility of autapomorphic SNPs yielding false positives for homoeologous conversion detection [8]. We found that homoeologous conversion detection was biased to favor dominant conversion events for the genome corresponding to the reference sequence used in the analysis. This suggests that many detected homoeologous conversions by SNPs may be due to artifacts of analysis and of imperfect data. Because of different genetic distances (A_2_ is closer to A_T_ and D_5_ is to D_T_) and completeness of reference sequences [19,20], false positive read mappings may have resulted in an overestimate of D-dominant homoeologous conversion events, as detected by both Guo et al. [11] and the current study.

In the CNV method, large blocks of homoeologous conversion manifest as duplication in one genome and deletion in the homoeologous region of the other genome. These events can be detected using the CNV method (duplication and deletion at homoeologous loci), although this detection suffers from increasing noise as the size of the sliding window is reduced, particularly under 1 kb. Overlapping duplication/deletion events have been detected in *Brassica* in whole genome sequencing data and their coverage patterns were attributed to non-reciprocal homoeologous recombination events [30]. These events detected by sequencing are reminiscent of chromosome rearrangements first observed by RFLP patterns in *B*. *napus* [6]. They are also likely recent events between the genomes because these large blocks of conversion have not been dissected by subsequent homologous recombination. In cotton, we did not detect clear support of any large blocks of homoeologous conversion. In addition, non-reciprocal homoeologous recombination has not been detected in cotton using genetic mapping technologies (RFLPs, SSRs, or SNPs) as it has in *Brassica* [6,7]. Perhaps, the block(s) on Chromosome 12 could be due to conversion events, but three pieces of independent evidence of conversion do not support it. While conversion events may occur frequently in other species, the size disparity between the A_T_- and D_T_-genomes may partly explain the lack of homoeologous conversion in cotton.

### Evolution of Tetraploid Species

Our results also cast light on the phylogenetic relationships among tetraploid species, including a newly characterized species, *G*. *ekmanianum* (AD_6_), and a possible new species of the Wake Island Atolls (AD_7_) [2,3]. Previous work had constructed cotton phylogenies based on select genes [31]. However, we use an unprecedented breadth and depth of data in cotton with SNPs from across the nuclear genome, resulting in over 48 million allele-SNPs. Other studies have disputed the species status of AD_6_ and suggested that it is merely wild AD_1_ [32]. However, our results show that AD_6_ and AD_7_ are both external to the wild AD_1_ accessions TX-0231 and TX-2094 and they are distant from the AD_1_ cultivars. AD_6_ and AD_7_ also form distinct clades that cannot be considered as one monophyletic species. We conclude that the species status for AD_6_ (*G*. *ekmanianum*) and proposed AD_7_ are supported by whole genome sequence data. PI-528167, although labeled an AD_2_ line, is clearly not AD_2_, as it consistently clusters within the AD_1_ clade. Genotypic (SSR) and phenotypic data also suggest that PI-528167 is a wild AD_1_ rather than AD_2_, corroborating this result (Richard Percy, personal communication).

These allele-SNPs form the beginning of a Cotton HapMap, similar to the database of SNPs constructed for the maize HapMap [16]. Our homoeo-SNP indices augment this database, resulting in a database of over 70 million SNPs among cotton species, though homoeo-SNPs are loci that researchers will want to avoid using for SNP-assays. The SNP data is organized according to their status as homoeo-SNPs between genome groups and allele-SNPs within genome groups. These SNPs are available for visualization and download on CottonGen [33] (http://www.cottongen.org/data/download).

### Domestication in Tetraploid Cotton

Artificial selection associated with domestication causes a genetic bottleneck in all domesticated plant species. This bottleneck results in cultivars having less genetic diversity compared to wild lines, as seen in WGS data of recent studies of soybean [34,35], tomato [17], pepper [36], bean [37], rice [38], and maize [39]. This phenomenon was observed in the AD_1_—and to a lesser degree AD_2_—cultivars, as manifested in the tight clustering of cultivars within the SNP-based phylogenetic trees. Small amounts of genetic diversity impose limits on the genetic potential of cotton breeding, since limited genetic diversity remained after domestication. Based on the WGS data produced in this study, significant genetic diversity exists in wild accessions of both *G*. *hirsutum* and *G*. *barbadense*. Some of the wild accessions sequenced here could be used for sources of additional genetic diversity in breeding programs. An effort to sequence all of the genetic diversity within cultivated and wild cotton accessions would provide a comprehensive perspective to inform genetic improvement of cotton.

Domestication increased the conservation of copy number variants (duplications and deletions) among cultivars as opposed to wild cotton lines. This is likely be a reflection of selection during domestication, and perhaps our small degree of sampling. Our study shows that A_T_-genome duplications were more (~2x) conserved than D_T_-genome duplications in AD_1_ cultivars, although not in AD_2_. While many fiber QTL are found in the A_T_-genome as well as the D_T_-genome [40], selection during domestication also appears to have favored A_T_-genome duplications. Also, A_T_-genome deletions were more conserved than D_T_-genome deletions in AD_2_ but not in AD_1_. Since our sampling of AD_2_ accessions were mostly wild, it’s unlikely that this conservation was caused by artificial selection for those deletions. Rather, these deletions likely occurred in the ancestral AD_2_ line that gave rise to the modern species, possibly contributing to the speciation and fiber quality that distinguish AD_2_ from other tetraploid cotton species. Both of these findings (greater numbers of duplications in A_T_ of AD_1_ and greater numbers of deletions in D_T_ of AD_2_) support the existence of independent domestication events for these two species.

Evidence of past attempts to introduce desirable traits from AD_1_ into AD_2_, or *vice versa*, was detected in the introgression detected in AD_1_ and AD_2_ cultivars (Figs 5 and 6). Some regions—including large regions of A_T_-Chr8 (Fig 5)—exhibited evidence of introgression in all AD_1_ cultivars, suggesting a relatively early event, while other, larger regions—e.g., D_T_-Chr09 (Fig 6)—evidenced introgression in a smaller number of cultivars, suggesting more recent introgression in the pedigrees of these lines. Breeders have long attempted to transfer genes for disease resistance, fiber quality, and other traits between AD_1_ and AD_2_, and we are now able to see genomic evidence of those efforts [5]. We also observed that an obsolete cultivar (Giza-7) had fewer genes commonly introgressed compared to other cultivars and a greater level of noise (*i*.*e*. fewer matched bases between wild AD_1_ and Giza-7 than other cultivars) suggesting less selection for agronomic improvement. In addition to introducing specific, targeted traits, new combinations of introgression may provide an additional source of diversity for the extremely narrow germplasm of cotton cultivars.

Resequencing the tetraploid genome of cotton provided insights into domestication, introgression, and homoeologous conversion in both *G*. *hirsutum* and *G*. *barbadense*. Our whole genome sequencing data supports the previously described independent domestication of these two polyploid species. The large genome-wide collection of SNPs between and within genomes provided an unprecedented examination of the single-nucleotide genetic diversity within the cotton genome, but a comprehensive assessment is not entirely complete. Additional re-sequencing of wild and domesticated cotton accessions will identify rare alleles, provide sufficient power for robust estimates of linkage disequilibrium (LD), and further identify regions of unique sequence evolution and domestication. Here, our limited sampling of both tetraploid species prohibited effective investigation of LD and selective sweeps. Nevertheless, this resequencing data provided important insights into the polyploid nature of the tetraploid cotton genome. Polyploidy has been a key aspect of cotton evolution and necessitates special computational consideration to properly use short read sequence data because of the close sequence similarity of homoeologs. In light of the large amount of genome sequence data, we found rare evidence for limited homoeologous exchanges, though no conclusive homoeologous exchanges were identified. In general, the sequences of cotton homoeologous loci have not significantly changed after polyploidization, though some exceptions can be found in individual gene pairs. Further research is needed to identify any association between these exceptions and the phenotype of modern cotton.

## Methods

Various components of BamBam (version 1.4) and SAMtools (version 1.2), along with custom scripts built on BioPerl, were used to modify, summarize, and analyze aligned sequence data throughout the processes described below [14,41,42].

### Sequence Data

In total, over 18 billion 100bp paired-end Illumina reads were generated by Huntsman Cancer Institute, BGI, University of California-Davis, and Mississippi State University across 33 accessions: 13 *G*. *hirsutum*, 15 *G*. *barbadense*, and 1 each of *G*. *tomentosum*, *G*. *mustelinum*, *G*. *darwinii*, *G*. *ekmanianum*, and 2 accessions from the Wake Island Atolls. Illumina sequence data for the diploids—3 *G*. *herbaceum*, 4 *G*. *arboreum*, and 4 *G*. *raimondii*—and one additional *G*. *hirsutum* were obtained from SRA. For *Gossypiodes kirkii*—an outgroup of the *Gossypium* genus—40 million 36 bp single-end Illumina reads were obtained from NCGR. Reads were trimmed with Sickle (https://github.com/najoshi/sickle) using a PHRED quality threshold of 20. Sequences used and generated in the effort are available in the Sequence Read Archive (S1 Table).

### Homoeo-SNP Identification, Mapping, and Read Categorization

Identification of homoeologous conversion events using short read data from cotton or other allopolyploid genera requires specialized software. We have identified and implemented two different strategies to categorize mapped reads from tetraploid cotton to their genome of origin: PolyCat [12] and PolyDog [13]. Both programs are freely available as part of BamBam [14] at https://sourceforge.net/projects/bambam/ and were used as part of this study. PolyCat uses GSNAP’s SNP-tolerant mapping with an index of known homoeo-SNPs (SNPs that differentiate the A and D genomes) instead of its traditional use to index SNPs in the human genome sequence. Consequently, the reads are aligned to a single diploid sequence (representing a relative of one of the parent genomes) with minimized mapping bias between genomes [15]. PolyCat then categorizes each tetraploid read to a genome (A_T_ or D_T_) based on whether it matches the A_T_- or D_T_-genome bases at previously known homoeo-SNP loci [12]. PolyDog maps the same set of polyploid reads to two different diploid reference sequences (*e*.*g*. one mapping analysis to an A-genome diploid reference and another mapping analysis to a D-genome diploid reference). Then PolyDog compares the quality of each read’s mapping to the different genome references and assigns the read to the genome that had a better mapping [18]. These two different approaches provide separate results that are used to address different, and sometimes complementary, biological questions.

The major difference between results produced by PolyCat and PolyDog is that PolyCat only categorizes reads that map over known or putatively identified homoeo-SNPs. Consequently, it only categorizes reads from regions that are present in both genomes. If a read originates in a region specific to the A_T_-genome (*i*.*e*., no D_T_-genome homoeolog exists), then that read cannot be formally SNP-categorized as originating in the A_T_- or D_T_-genome. On the other hand, PolyDog can categorize reads virtually anywhere in the genome. In practice, this means that PolyDog categorizes more reads and produces a smoother coverage profile over more of the genome, while PolyCat produces islands of homoeologous coverage separated by regions that are either identical between genomes or specific to one genome or another [18]. PolyCat has a lower error rate than PolyDog and is preferred for situations in which the presence of genome-specific regions causes additional biases in the mapping results. PolyCat-categorized reads are all mapped to a single reference, allowing straightforward comparisons between A_T_ and D_T_ reads in regions of homoeology, particularly in areas of sequence conservation (*e*.*g*. genes). PolyDog-categorized reads are mapped to two different references, making it difficult to perform direct homoeologous comparisons at a single nucleotide resolution.

The primary alternative to read categorization methods is mapping reads to a ‘full’ reference sequence representing both genomes of tetraploid cotton, whether that sequence is a concatenation of two diploid genome sequences [20] or a *de novo* assembly of a tetraploid cotton [20,21]. This mapping approach is comparable to PolyDog, as it maps reads anywhere in the genome rather than only to homoeologous regions. As shown previously and in this study, the PolyDog method accurately maps (and categorizes) more reads to the two diploid references than traditional read mapping to the ‘full’ reference sequence method [18]. We primarily use PolyDog-categorized reads in this study, employing PolyCat only where it is necessary either to reduce the area in question to homoeologous regions or to directly compare homoeologs at a specific nucleotide position.

All reads were aligned to both the D_5_ and A_2_ reference genomes with GSNAP using the options “-n 1 –Q” to require unique best mappings [15,20]. An index of homoeo-SNPs inferred from diploid whole-genome resequencing was used for GSNAP SNP-tolerant mapping (“-v” option) [18]. Reads were then categorized as originating in the A_T_- or D_T_-genome by PolyCat, using a diploid-based homoeo-SNP index. Briefly, the homoeo-SNP index was constructed by mapping reads from both diploids species to the ‘other’ genome reference (*e*.*g*. A-genome reads to D-genome reference). SNPs between genomes were then identified and compiled into a SNP-index for GSNAP. The original diploid reads were then re-mapped (*e*.*g*. A-genome reads to D-genome reference with SNP-tolerant mapping). In this second iteration, more reads were mapped because this time, reads were not penalized by mismatching SNPs during mapping. In addition, new SNPs between genomes were identified because now more reads were mapping to the reference. These new SNPs were added to this putative homoeo-SNP index. The process was repeated until no new putative homoeo-SNPs were found between diploids. Then reads from the tetraploid were mapped using the diploid SNP-index. Mapped reads overlapping putative homoeo-SNPs confirmed SNPs as homoeo-SNPs (or not). The tetraploid reads were then categorized to the A_T_ or D_T_-genome based on nucleotide matches at SNP loci. If the tetraploid base matched the A_2_-genome base, then read was categorized at A_T_. Some new homoeo-SNPs were discovered that were specific for the tetraploid genome A_2_ and D_5_ are not the actual genome ancestors of tetraploid cotton. These new tetraploid-specific homoeo-SNPs where also added to the SNP-index. Like the diploid reads, tetraploid reads were iteratively re-mapped to the diploid reference to identify additional homoeo-SNPs until no new homoeo-SNPs were found. This iterative process was repeated for each species so that each species has its own SNP-index.

InterSnp (part of BamBam) was used to call SNPs between individuals with a minimum allele coverage of 5 reads per individual, and SNPs that consistently (75% of observed genotypes) manifested in one genome of a species—and were consistently (75%) absent in the other genome of that species—were called as homoeo-SNPs [26]. Only 1 accession each of AD_3_, AD_4_, and AD_5_ were available (and these species have sufficiently narrow germplasm that one accession is a fair sampling of the species), so a 100% threshold was used, rather than 75%. Five tetraploid-based homoeo-SNP indices were then generated for each genome, one each for AD_1_, AD_2_, AD_3_, AD_4_, and AD_5_, named D13.snp4.1 through D13.snp4.5 (or A13.snp2.1 through A13.snp2.5), respectively. We also made modified reference sequences for each genome of each tetraploid species by replacing the ancestral nucleotide with that indicated by the homoeo-SNP index. The newly identified species AD_6_ and AD_7_ are very closely related to AD_1_ (as shown below), so mappings to AD_6_ and AD_7_ use the AD_1_-based homoeo-SNP indices and modified reference sequences. To estimate the number of SNPs between homoeologs, best-hits of reciprocal BLAST were used to establish a list of homoeologs A_T_-D_T_ pairs [43].

Indel-induced mapping errors were corrected using GATK [44]. First, RealignerTargetCreator was run on a group of 20 A_T_-genome BAM files and on 20 D_T_-genome BAM files (representing all tetraploid species). Second, IndelRealigner was used on each individual BAM file to adjust read alignments around the indels identified in the first step: 3,692,540 loci in the A_2_ reference and 2,195,978 loci in the D_5_ reference.

### Single Nucleotide Polymorphisms

SNPs and short indels were called—once for all A_T_-genome BAM files and once for all D_T_-genome BAM files—between the PolyDog-categorized genomes using InterSnp with a minimum coverage per allele of 5 reads and minimum frequency of 30% [14]. A neighbor-joining tree was constructed for each genome, bootstrapping 1000 sub-samples without replacement with 5% of SNPs in each sub-sample. Trees were generated by creating a distance matrix based on genotypes at all SNP loci, then running neighbor (from PHYLIP) with random sample ordering to build the actual tree [45]. The 1000 trees from the bootstraps were combined with consense (from PHYLIP) to make a single consensus tree. Trees were visualized in Geneious [29].

Small homoeologous conversions were analyzed by using PolyCat to categorize mapped reads from each tetraploid because PolyCat categorization allows for inter-genomic analysis at a nucleotide level [12]. Then SNPs were called with InterSnp across all species and genomes [26]. Consensus genotypes were called for each species at sites that had coverage from at least 75% of individuals (10/13 for AD_1_ and 11/14 for AD_2_), and genotype patterns suggestive of homoeologous conversion in AD_1_ or AD_2_ were identified (*e*.*g*., A_2_, A_T_, and D_T_ have a C while D_5_ has a T).

### Copy Number Variants

Copy number variants (CNVs) were called in the PolyDog-categorized A_T_- and D_T_-genomes of each sample, relative to their respective diploid relatives, using CNVKit [30]. Reads from 3 diploid A_2_ lines and 4 diploid D_5_ lines were mapped and categorized in the same manner as the reads from the tetraploids, providing reference coverage profiles for the A- and D-genomes, which serve to normalize for biases in sequence coverage that are shared between diploid and tetraploid members of a common genome. The coverage of each tetraploid genome was compared to the reference coverage profile of its diploid relative. The gene annotations for each reference sequence were provided as targets, and accessible regions of the genome were identified for filtering by a CNVKit utility script genome2access.py. Segments identified by CNVKit as having a log base 2 copy number of at least 1.0 were considered duplications in the tetraploid genome, and segments identified with a log base 2 copy number of -1.0 or less were considered deletions.

## Supporting Information

S1 Table Number of reads and amount of coverage, along with mapping and categorization rates for each library (See Excel file). (XLSX)

S2 Table Copy number variants (duplications and deletions) in each tetraploid cotton line (See Excel file). (XLSX)

S3 Table Conserved copy number variants across sub-groups of tetraploids. Few genes were duplicated or deleted in several different accessions.
(DOCX)

S4 Table Ancient gene conversion events based on SNP patterns in diploids and tetraploid genomes. (DOCX)

S5 Table Genes in possible large homoeologous conversion events by accession (A) and by gene (B).
(DOCX)

S6 Table D-statistics for tests of introgression between AD_1_ and AD_2_ cultivars. Introgression was more evident in genes than in the genome at large, consistent with introgression from breeding efforts. D-statistics were much higher in putative introgressed regions, validating the methodology for identifying introgressed regions.
(DOCX)

S1 Fig Diversity sliding window. For each chromosome, a Fig shows diversity levels in a sliding window 100 Kbp wide stepping by 50 Kbp. Nucleotide positions are shown at the bottom of each plot. The dark blue line shows the number of SNPs per base pair (bp) found among all members of that genome group (A or D), including diploids. Plots labeled ‘Chr1’ are mapped against the D_5_ genome reference sequence [20]. Charts labeled ‘Chr01’ represent reads mapped against the A_2_ genome reference sequence [21]. Note also that the D5 The red line is SNPs/bp among tetraploids only. The green line is SNPs/bp among members of AD_1_, AD_6_, and AD_7_. The purple line is SNPs/bp among AD_1_ cultivars. The light blue line is SNPs/bp among members of AD_2_.
(PDF)

S2 Fig Lengths of 26,782 homoeolog gene pairs were highly correlated (Pearson *r*^2^ = 0.744, *p*-value < 2.2e-16) as identified by BLAST [43]. The density of allele-SNPs was weakly correlated among allotetraploids (Pearson *r*^2^ = 0.321, *p*-value < 2.2e^-16^; Supp. Fig 2A) and among AD_1_ cultivars (Pearson *r*^2^ = 0.261, *p*-value < 2.2e^-16^; Supp. Fig 2B).
(PDF)

S3 Fig Exceptional gene histogram. The number of SNPs in one gene when its homoeolog has 0 SNPs. Red is for genes that have 0 SNPs in the A_T_-genome homoeolog; green is for genes that have 0 SNPs in the D_T_-genome homoeolog. To identify homoeolog pairs in the annotations of the A_2_ and D_5_ reference sequences, we used BLASTP with a maximum e-value of 10^−20^ to compare the peptide sequences of annotated A_2_ and D_5_ genes [43].
(PDF)

## References

[1] Wendel JF. New World tetraploid cottons contain Old World cytoplasm. PNAS. 1989;86: 4132–4136. 16594050 10.1073/pnas.86.11.4132 PMC287403

[2] Krapovickas A, Seijo G. *Gossypium ekmanianum* (*Malvaceae*), algodon silvestre de la Republica Dominicana. Bonplandia. 2008;17: 55–63.

[3] Grover CE, Zhu X, Grupp KK, Jareczek JJ, Gallagher JP, Szadkowski E, et al Molecular confirmation of species status for the allopolyploid cotton species, *Gossypium ekmanianum* Wittmack. Genet Resour Crop Evol. Springer Netherlands; 2014;62: 1–12. 10.1007/s10722-014-0138-x

[4] Grover CE, Grupp KK, Wanzek RJ, Wendel JF. Assessing the monophyly of polyploid *Gossypium* species. Plant Syst Evol. Springer Vienna; 2012;298: 1177–1183. 10.1007/s00606-012-0615-7

[5] Wendel JF, Cronn RC. Polyploidy and the evolutionary history of cotton. Advances in Agronomy. Elsevier; 2003 pp. 139–186. 10.1016/S0065-2113(02)78004-8

[6] Udall JA, Quijada PA, Osborn TC. Detection of chromosomal rearrangements derived from homologous recombination in four mapping populations of *Brassica napus* L. Genetics. 2005;169: 967–979. 10.1534/genetics.104.033209 15520255 PMC1449096

[7] Sharpe AG, Parkin IA, Keith DJ, Lydiate DJ. Frequent nonreciprocal translocations in the amphidiploid genome of oilseed rape (*Brassica napus*). Genome. 1995;38: 1112–1121. 18470235 10.1139/g95-148

[8] Flagel LE, Wendel JF, Udall JA. Duplicate gene evolution, homoeologous recombination, and transcriptome characterization in allopolyploid cotton. BMC Genomics. BMC Genomics; 2012;13: 1–1. 22768919 10.1186/1471-2164-13-302 PMC3427041

[9] Salmon A, Flagel L, Ying B, Udall JA, Wendel JF. Homoeologous nonreciprocal recombination in polyploid cotton. New Phytologist. 2010;186: 123–134. 10.1111/j.1469-8137.2009.03093.x 19925554

[10] Gaeta RT, Pires JC. Homoeologous recombination in allopolyploids: the polyploid ratchet. New Phytologist. 2010;186: 18–28. 10.1111/j.1469-8137.2009.03089.x 20002315

[11] Guo H, Wang X, Gundlach H, Mayer KFX, Peterson DG, Scheffler BE, et al Extensive and biased intergenomic non-reciprocal DNA exchanges shaped a nascent polyploid genome, *Gossypium* (cotton). Genetics. Genetics Society of America; 2014;197: 1153–1163. 10.1534/genetics.114.166124 24907262 PMC4125390

[12] Page JT, Gingle AR, Udall JA. PolyCat: A resource for genome categorization of sequencing reads from allopolyploid organisms. G3: Genes|Genomes|Genetics. 2013;3: 517–525. 10.1534/g3.112.005298 23450226 PMC3583458

[13] Page JT, Udall JA. Methods for mapping and categorization of DNA sequence reads from allopolyploid organisms. BMC Genetics. BioMed Central Ltd; 2015;16: S4 10.1186/1471-2156-16-S2-S4 25951770 PMC4423573

[14] Page JT, Liechty ZS, Huynh MD, Udall JA. BamBam: genome sequence analysis tools for biologists. BMC Research Notes 2014 7:1. BioMed Central; 2014;7: 1. 25421351 10.1186/1756-0500-7-829 PMC4258253

[15] Wu TD, Nacu S. Fast and SNP-tolerant detection of complex variants and splicing in short reads. Bioinformatics. 2010;26: 873–881. 10.1093/bioinformatics/btq057 20147302 PMC2844994

[16] Chia J-M, Song C, Bradbury PJ, Costich D, de Leon N, Doebley J, et al Maize HapMap2 identifies extant variation from a genome in flux. Nat Genet. Nature Publishing Group; 2012;44: 803–807. 10.1038/ng.2313 22660545

[17] Lin T, Zhu G, Zhang J, Xu X, Yu Q, Zheng Z, et al Genomic analyses provide insights into the history of tomato breeding. Nature Publishing Group. Nature Publishing Group; 2014;46: 1220–1226. 10.1038/ng.3117 25305757

[18] Page JT, Huynh MD, Liechty ZS, Grupp K, Stelly D, Hulse AM, et al Insights into the evolution of cotton diploids and polyploids from whole-genome re-sequencing. G3. Genetics Society of America; 2013;3: 1809–1818. 10.1534/g3.113.007229 23979935 PMC3789805

[19] Li F, Fan G, Wang K, Sun F, Yuan Y, Song G, et al Genome sequence of the cultivated cotton *Gossypium arboreum*. Nat Genet. Nature Publishing Group; 2014;46: 567–572. 10.1038/ng.2987 24836287

[20] Paterson AH, Wendel JF, Gundlach H, Guo H, Jenkins J, Jin D, et al Repeated polyploidization of *Gossypium* genomes and the evolution of spinnable cotton fibres. Nature Publishing Group; 2012;492: 423–427. 10.1038/nature11798 23257886

[21] Li F, Fan G, Lu C, Xiao G, Zou C, Kohel RJ, et al Genome sequence of cultivated Upland cotton (*Gossypium hirsutum* TM-1) provides insights into genome evolution. Nat Biotechnol. 2015;33: 524–530. 10.1038/nbt.3208 25893780

[22] Zhang T, Hu Y, Jiang W, Fang L, Guan X, Chen J, et al Sequencing of allotetraploid cotton (*Gossypium hirsutum* L. acc. TM-1) provides a resource for fiber improvement. Nat Biotechnol. Nature Publishing Group; 2015;33: 531–537. 10.1038/nbt.3207 25893781

[23] Zhao M, Wang Q, Wang Q, Jia P, Zhao Z. Computational tools for copy number variation (CNV) detection using next-generation sequencing data: features and perspectives. BMC bioinformatics. BioMed Central Ltd; 2013;14: S1 10.1186/1471-2105-14-S11-S1 PMC3846878 24564169

[24] Talevich E., Shain A.H., Botton T., & Bastian B.C. (2014). CNVkit: Copy number detection and visualization for targeted sequencing using off-target reads. bioRxiv 10.1101/010876 PMC4839673 27100738

[25] Chaudhary B, Hovav R, Rapp R, Verma N, Udall JA, Wendel JF. Global analysis of gene expression in cotton fibers from wild and domesticated *Gossypium barbadense*. Evol Dev. 2008;10: 567–582. 10.1111/j.1525-142X.2008.00272.x 18803775

[26] Wendel JF, Grover CE. Taxonomy and evolution of the cotton genus, *Gossypium*. Cotton. American Society of Agronomy, Inc., Crop Science Society of America, Inc., and Soil Science Society of America, Inc; 2015 pp. 25–44. 10.2134/agronmonogr57.2013.0020

[27] Wendel JF, Grover CE. Taxonomy and evolution of the cotton genus, *Gossypium*. Cotton. American Society of Agronomy, Inc., Crop Science Society of America, Inc., and Soil Science Society of America, Inc; 2015 pp. 25–44. 10.2134/agronmonogr57.2013.0020

[28] Quinlan AR, Hall IM. BEDTools: a flexible suite of utilities for comparing genomic features. Bioinformatics. 2010;26: 841–842. 10.1093/bioinformatics/btq033 20110278 PMC2832824

[29] Durand EY, Patterson N, Reich D, Slatkin M. Testing for ancient admixture between closely related populations. Molecular Biology and Evolution. Oxford University Press; 2011;28: 2239–2252. 10.1093/molbev/msr048 21325092 PMC3144383

[30] Chalhoub B, Denoeud F, Liu S, Parkin IAP, Tang H, Wang X, et al Plant genetics. Early allopolyploid evolution in the post-Neolithic *Brassica napus* oilseed genome. Science. American Association for the Advancement of Science; 2014;345: 950–953. 10.1126/science.1253435 25146293

[31] Senchina DS, Alverez I, Cronn RC, Liu B, Rong J, Noyes RD, et al Rate variation among nuclear genes and the age of polyploidy in gossypium. Molecular Biology and Evolution. 2003;20: 633–643. 10.1093/molbev/msg065 12679546

[32] d'Eeckenbrugge GC, Lacape J-M. Distribution and Differentiation of Wild, Feral, and Cultivated Populations of Perennial Upland Cotton (*Gossypium hirsutum* L.) in Mesoamerica and the Caribbean. Zhang X, editor. PLoS ONE. Public Library of Science; 2014;9: e107458 10.1371/journal.pone.0107458 25198534 PMC4157874

[33] Yu J, Jung S, Cheng CH, Ficklin SP, Lee T, Zheng P, et al CottonGen: a genomics, genetics and breeding database for cotton research. Nucleic Acids Research. 2013 10.1093/nar/gkt1064 PMC3964939 24203703

[34] Zhou Z, Jiang Y, Wang Z, Gou Z, Lyu J, Li W, et al Resequencing 302 wild and cultivated accessions identifies genes related to domestication and improvement in soybean. Nat Biotechnol. 2015;33: 408–414. 10.1038/nbt.3096 25643055

[35] Lam H-M, Xu X, Liu X, Chen W, Yang G, Wong F-L, et al Resequencing of 31 wild and cultivated soybean genomes identifies patterns of genetic diversity and selection. Nat Genet. 2010;42: 1053–1059. 10.1038/ng.715 21076406

[36] Qin C, Yu C, Shen Y, Fang X, Chen L, Min J, et al Whole-genome sequencing of cultivated and wild peppers provides insights into *Capsicum* domestication and specialization. PNAS. National Acad Sciences; 2014;: 201400975 10.1073/pnas.1400975111 PMC3986200 24591624

[37] Schmutz J, McClean PE, Mamidi S, Wu GA, Cannon SB, Grimwood J, et al A reference genome for common bean and genome-wide analysis of dual domestications. Nat Genet. 2014;46: 707–713. 10.1038/ng.3008 24908249 PMC7048698

[38] Huang X, Kurata N, Wei X, Wang Z-X, Wang A, Zhao Q, et al A map of rice genome variation reveals the origin of cultivated rice Nature Publishing Group; 2012;:–. 10.1038/nature11532 PMC7518720 23034647

[39] Hufford MB, Xu X, van Heerwaarden J, Pyhäjärvi T, Chia J-M, Cartwright RA, et al Comparative population genomics of maize domestication and improvement. Nat Genet. Nature Publishing Group; 2012;44: 808–811. 10.1038/ng.2309 22660546 PMC5531767

[40] Rong J, Feltus FA, Waghmare VN, Pierce GJ, Chee PW, Draye X, et al Meta-analysis of Polyploid cotton QTL shows unequal contributions of subgenomes to a complex network of genes and gene clusters implicated in lint fiber development. Genetics. Genetics; 2007;176: 2577–2588. 10.1534/genetics.107.074518 17565937 PMC1950656

[41] Stajich JE, Block D, Boulez K, Brenner SE, Chervitz SA, Dagdigian C, et al The Bioperl toolkit: Perl modules for the life sciences. Genome Res. 2002;12: 1611–1618. 10.1101/gr.361602 12368254 PMC187536

[42] Li H, Handsaker B, Wysoker A, Fennell T, Ruan J, Homer N, et al The Sequence Alignment/Map format and SAMtools. Bioinformatics 2009;25: 2078–2079. 10.1093/bioinformatics/btp352 19505943 PMC2723002

[43] Altschul SF, Gish W, Miller W, Myers EW, Lipman DJ. Basic local alignment search tool. J Mol Biol. 1990;215: 403–410. 10.1016/S0022-2836(05)80360-2 2231712

[44] DePristo MA, Banks E, Poplin R, Garimella KV, Maguire JR, Hartl C, et al A framework for variation discovery and genotyping using next-generation DNA sequencing data. Nat Genet. 2011;43: 491–498. 10.1038/ng.806 21478889 PMC3083463

[45] Felsenstein J. PHYLIP—Phylogeny Inference Package (Version 3.2). Cladistics. Blackwell Publishing Ltd; 1989;5: 163–166. 10.1111/j.1096-0031.1989.tb00562.x

