# Correction: DNA Sequence Evolution and Rare Homoeologous Conversion in Tetraploid Cotton

**DOI:** 10.1371/journal.pgen.1006206

**Published:** 2016-07-22

**Authors:** Justin T. Page, Zach S. Liechty, Rich H. Alexander, Kimberly Clemons, Amanda M. Hulse-Kemp, Hamid Ashrafi, Allen Van Deynze, David M. Stelly, Joshua A. Udall

## Notice of Republication

S1 Table and S2 Table were published in error. The errors were corrected in the HTML and PDF versions of the article on June 22, 2016, and the corrected tables are available to view in the supporting information of this article.

## Supporting Information

S1 File Corrected S1 Table (XLSX)

S2 File Corrected S2 Table (XLSX)
