# Supplementary material for: DNA Sequence Evolution and Rare Homoeologous Conversion in Tetraploid Cotton
Source: PLoS Genet. 2016 May 11;12(5):e1006012. doi: 10.1371/journal.pgen.1006012 (PMC4864293; doi:10.1371/journal.pgen.1006012)
Supplement: S3 Table — Few genes were duplicated or deleted in several different accessions. (DOCX) [file pgen.1006012.s003.docx]

# Supporting Information

S3 Table Conserved copy number variants across sub-groups of tetraploids. Few genes were duplicated or deleted in several different accessions.

| **Type** | **Group** | **Total Genes** | **Found in at least 50% of accessions** | |
| --- | --- | --- | --- | --- |
| A-duplication | AD_1_-domesticates | 193 | 34 | 18% |
|  | AD_1_-clade | 258 | 30 | 12% |
|  | All | 307 | 21 | 7% |
| D-duplication | AD_1_-domesticates | 188 | 24 | 13% |
|  | AD_1_-clade | 286 | 20 | 7% |
|  | All | 387 | 21 | 5% |
| A-deletion | AD_1_-domesticates | 785 | 405 | 52% |
|  | AD_1_-clade | 937 | 385 | 41% |
|  | All | 1539 | 369 | 24% |
| D-deletion | AD_1_-domesticates | 604 | 273 | 45% |
|  | AD_1_-clade | 764 | 216 | 28% |
|  | All | 1114 | 196 | 18% |
