# Supplementary material for: DNA Sequence Evolution and Rare Homoeologous Conversion in Tetraploid Cotton
Source: PLoS Genet. 2016 May 11;12(5):e1006012. doi: 10.1371/journal.pgen.1006012 (PMC4864293; doi:10.1371/journal.pgen.1006012)
Supplement: S4 Table — (DOCX) [file pgen.1006012.s004.docx]

S4 Table Ancient gene conversion events based on SNP patterns in diploids and tetraploid genomes.

| **Species** |  |  |  |  | **Present in** |  |
| --- | --- | --- | --- | --- | --- | --- |
| **A_2_-reference** | **AD_1_** |  | **AD_2_** |  | **Both AD_1_ and AD_2_** |  |
| Autapamorphy in A_2_ | 605,147 | 36.1% | 605,514 | 36.5% | 599,400 | 37.4% |
| Autapamorphy in D_5_ | 1,005,869 | 60.1% | 996,741 | 60.1% | 977,987 | 61.1% |
| A-dominant Conversion | 41,759 | 2.5% | 37,960 | 2.3% | 17,130 | 1.1% |
| D-dominant Conversion | 21,593 | 1.3% | 19,493 | 1.2% | 6,406 | 0.4% |
|  |  |  |  |  |  |  |
| **D_5_-reference** |  |  |  |  |  |  |
| Autapamorphy in A_2_ | 913,769 | 39.7% | 912,441 | 40.0% | 900,332 | 41.2% |
| Autapamorphy in D_5_ | 1,270,268 | 55.2% | 1,259,994 | 55.2% | 1,235,851 | 56.5% |
| A-dominant Conversion | 52,680 | 2.3% | 50,528 | 2.2% | 21,857 | 1.0% |
| D-dominant Conversion | 65,276 | 2.8% | 58,898 | 2.6% | 27,757 | 1.3% |
