# Supplementary material for: DNA Sequence Evolution and Rare Homoeologous Conversion in Tetraploid Cotton
Source: PLoS Genet. 2016 May 11;12(5):e1006012. doi: 10.1371/journal.pgen.1006012 (PMC4864293; doi:10.1371/journal.pgen.1006012)
Supplement: S5 Table — Genes in possible large homoeologous conversion events by accession (A) and by gene (B). (DOCX) [file pgen.1006012.s005.docx]

S5 Table Genes in possible large homoeologous conversion events by accession (A) and by gene (B).

| **A)** | **Accessions** | **# Genes** |  | **B)** | **D5 gene** | **A2 gene** | **# Accessions** |
| --- | --- | --- | --- | --- | --- | --- | --- |
|  | AD3 | 15 |  |  | Gorai.012G000600 | Cotton_A_20329 | 14 |
|  | AD5 | 16 |  |  | Gorai.012G000700 | Cotton_A_20330 | 14 |
|  | AD7 | 17 |  |  | Gorai.012G000900 | Cotton_A_20331 | 14 |
|  | DP340 | 15 |  |  | Gorai.012G001000 | Cotton_A_20332 | 14 |
|  | FM832 | 17 |  |  | Gorai.012G001100 | Cotton_A_20334 | 14 |
|  | GB287 | 15 |  |  | Gorai.012G001200 | Cotton_A_20335 | 14 |
|  | GB362 | 15 |  |  | Gorai.012G001500 | Cotton_A_20338 | 14 |
|  | GB398 | 15 |  |  | Gorai.012G001600 | Cotton_A_20339 | 14 |
|  | GB618 | 15 |  |  | Gorai.012G001700 | Cotton_A_20340 | 14 |
|  | GB67 | 16 |  |  | Gorai.012G001800 | Cotton_A_20341 | 14 |
|  | MS240 | 15 |  |  | Gorai.012G001900 | Cotton_A_20342 | 14 |
|  | Phy76 | 15 |  |  | Gorai.012G002000 | Cotton_A_20343 | 14 |
|  | SG747 | 15 |  |  | Gorai.012G002100 | Cotton_A_20344 | 14 |
|  | TX321 | 15 |  |  | Gorai.012G002200 | Cotton_A_20345 | 14 |
|  | Maxxa | 2 |  |  | Gorai.012G002300 | Cotton_A_20346 | 14 |
|  |  |  |  |  | Gorai.012G000400 | Cotton_A_33577 | 1 |
|  |  |  |  |  | Gorai.007G329600 | Cotton_A_35502 | 3 |
|  |  |  |  |  | Gorai.007G329800 | Cotton_A_35503 | 3 |
|  |  |  |  |  | Gorai.001G162500 | Cotton_A_10073 | 1 |
|  |  |  |  |  | Gorai.002G268400 | Cotton_A_00274 | 1 |
|  |  |  |  |  | Gorai.002G268600 | Cotton_A_00273 | 1 |
