# Supplementary material for: DNA Sequence Evolution and Rare Homoeologous Conversion in Tetraploid Cotton
Source: PLoS Genet. 2016 May 11;12(5):e1006012. doi: 10.1371/journal.pgen.1006012 (PMC4864293; doi:10.1371/journal.pgen.1006012)
Supplement: S6 Table — Introgression was more evident in genes than in the genome at large, consistent with introgression from breeding efforts. D-statistics were much higher in putative introgressed regions, validating the methodology for identifying introgressed regions. (DOCX) [file pgen.1006012.s006.docx]

S6 Table D-statistics for tests of introgression between AD_1_ and AD_2_ cultivars. Introgression was more evident in genes than in the genome at large, consistent with introgression from breeding efforts. D-statistics were much higher in putative introgressed regions, validating the methodology for identifying introgressed regions.

|  |  | **Maxxa.A** | **Maxxa.D** | **TM1.A** | **TM1.D** | **Coker.A** | **Coker.D** | **Tamcot.A** | **Tamcot.D** | **Phy76.A** | **Phy76.D** | **DP340.A** | **DP340.D** |
| --- | --- | --- | --- | --- | --- | --- | --- | --- | --- | --- | --- | --- | --- |
| **Genomic** | "BABA" sites | 20,696 | 23,683 | 18,071 | 21,426 | 17,582 | 21,227 | 19,524 | 55,240 | 51,652 | 31,428 | 53,643 | 31,128 |
|  | "ABBA" sites | 11,205 | 16,870 | 11,012 | 17,944 | 11,299 | 18,079 | 11,864 | 16,958 | 4,670 | 11,948 | 4,724 | 12,345 |
|  | D-statistic | 0.30 | 0.17 | 0.24 | 0.09 | 0.22 | 0.08 | 0.24 | 0.53 | 0.83 | 0.45 | 0.84 | 0.43 |
| **Genic** | "BABA" sites | 2,183 | 3,741 | 1,760 | 3,301 | 1,606 | 3,223 | 2,141 | 5,883 | 4,058 | 4,183 | 3,936 | 4,272 |
|  | "ABBA" sites | 922 | 2,291 | 897 | 2,361 | 926 | 2,426 | 1,014 | 2,482 | 436 | 2,115 | 414 | 2,273 |
|  | D-statistic | 0.41 | 0.24 | 0.32 | 0.17 | 0.27 | 0.14 | 0.36 | 0.41 | 0.81 | 0.33 | 0.81 | 0.31 |
| **Introgression** | "BABA" sites | 3,482 | 8,434 | 1,767 | 5,579 | 1,429 | 5,498 | 2,822 | 29,454 | 31,859 | 15,105 | 35,366 | 16,038 |
|  | "ABBA" sites | 382 | 171 | 239 | 183 | 211 | 158 | 350 | 158 | 344 | 262 | 329 | 310 |
|  | D-statistic | 0.80 | 0.96 | 0.76 | 0.94 | 0.74 | 0.94 | 0.78 | 0.99 | 0.98 | 0.97 | 0.98 | 0.96 |
