# Supplementary material for: DNA Sequence Evolution and Rare Homoeologous Conversion in Tetraploid Cotton
Source: PLoS Genet. 2016 May 11;12(5):e1006012. doi: 10.1371/journal.pgen.1006012 (PMC4864293; doi:10.1371/journal.pgen.1006012)
Supplement: S1 Fig — For each chromosome, a Fig shows diversity levels in a sliding window 100 Kbp wide stepping by 50 Kbp. Nucleotide positions are shown at the bottom of each plot. The dark blue line shows the number of SNPs per base pair (bp) found among all members of that genome group (A or D), including diploids. Plots labeled ‘Chr1’ are mapped against the D5 genome reference sequence [20]. Charts labeled ‘Chr01’ represent reads mapped against the A2 genome reference sequence [21]. Note also that the D5 The red line is SNPs/bp among tetraploids only. The green line is SNPs/bp among members of AD1, AD6, and AD7. The purple line is SNPs/bp among AD1 cultivars. The light blue line is SNPs/bp among members of AD2. (PDF) [file pgen.1006012.s007.pdf]

S1 Fig

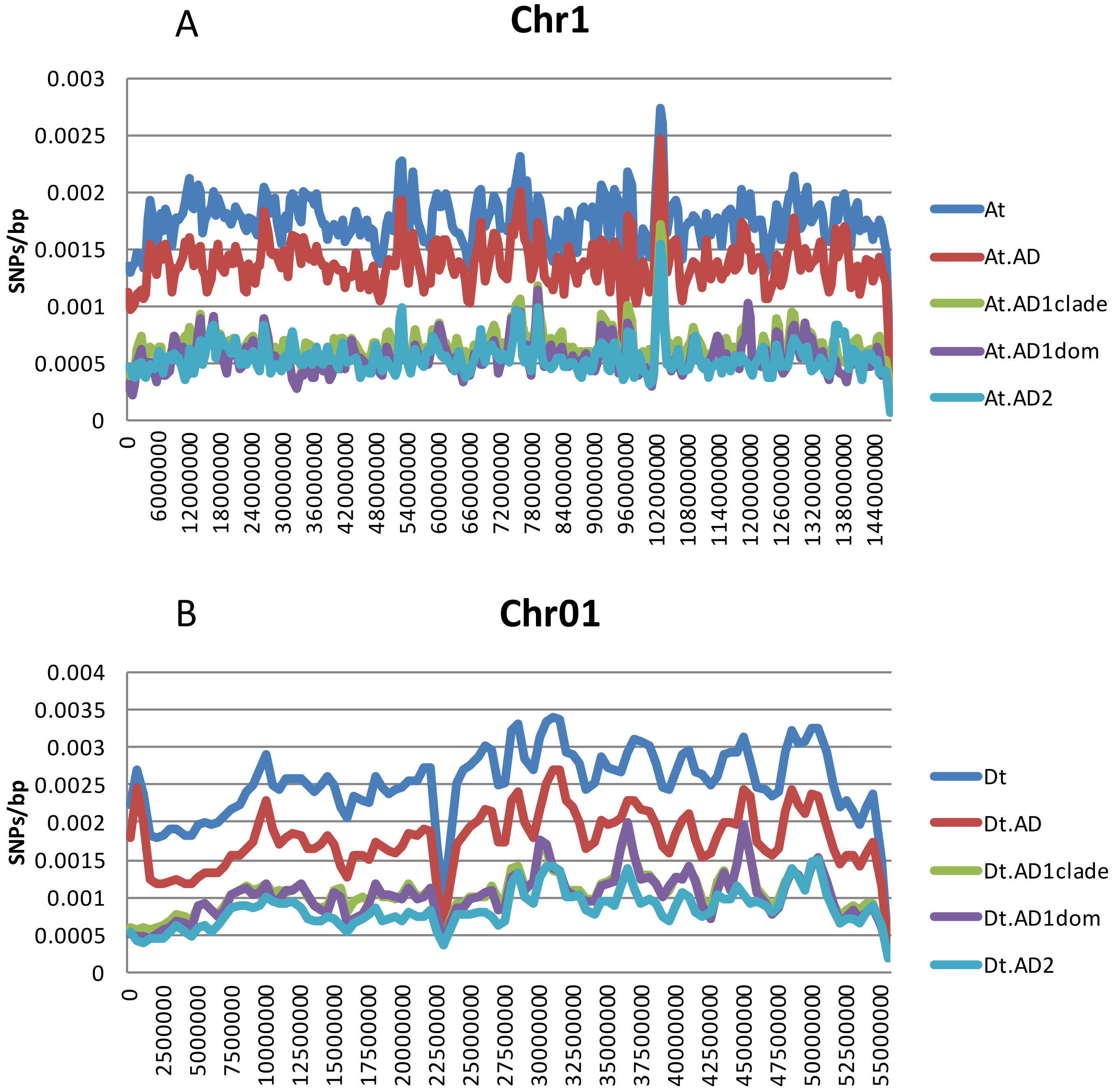
A
Chr1
SNPs/bp
0.003
0.0025
0.002
0.0015
0.001
0.0005
0
0
6000000
12000000
18000000
24000000
30000000
36000000
42000000
48000000
54000000
60000000
66000000
72000000
78000000
84000000
90000000
96000000
102000000
108000000
114000000
120000000
126000000
132000000
138000000
144000000
At
At.AD
At.AD1clade
At.AD1dom
At.AD2
B
Chr01
SNPs/bp
0.004
0.0035
0.003
0.0025
0.002
0.0015
0.001
0.0005
0
0
2500000
5000000
7500000
10000000
12500000
15000000
17500000
20000000
22500000
25000000
27500000
30000000
32500000
35000000
37500000
40000000
42500000
45000000
47500000
50000000
52500000
55000000
Dt
Dt.AD
Dt.AD1clade
Dt.AD1dom
Dt.AD2

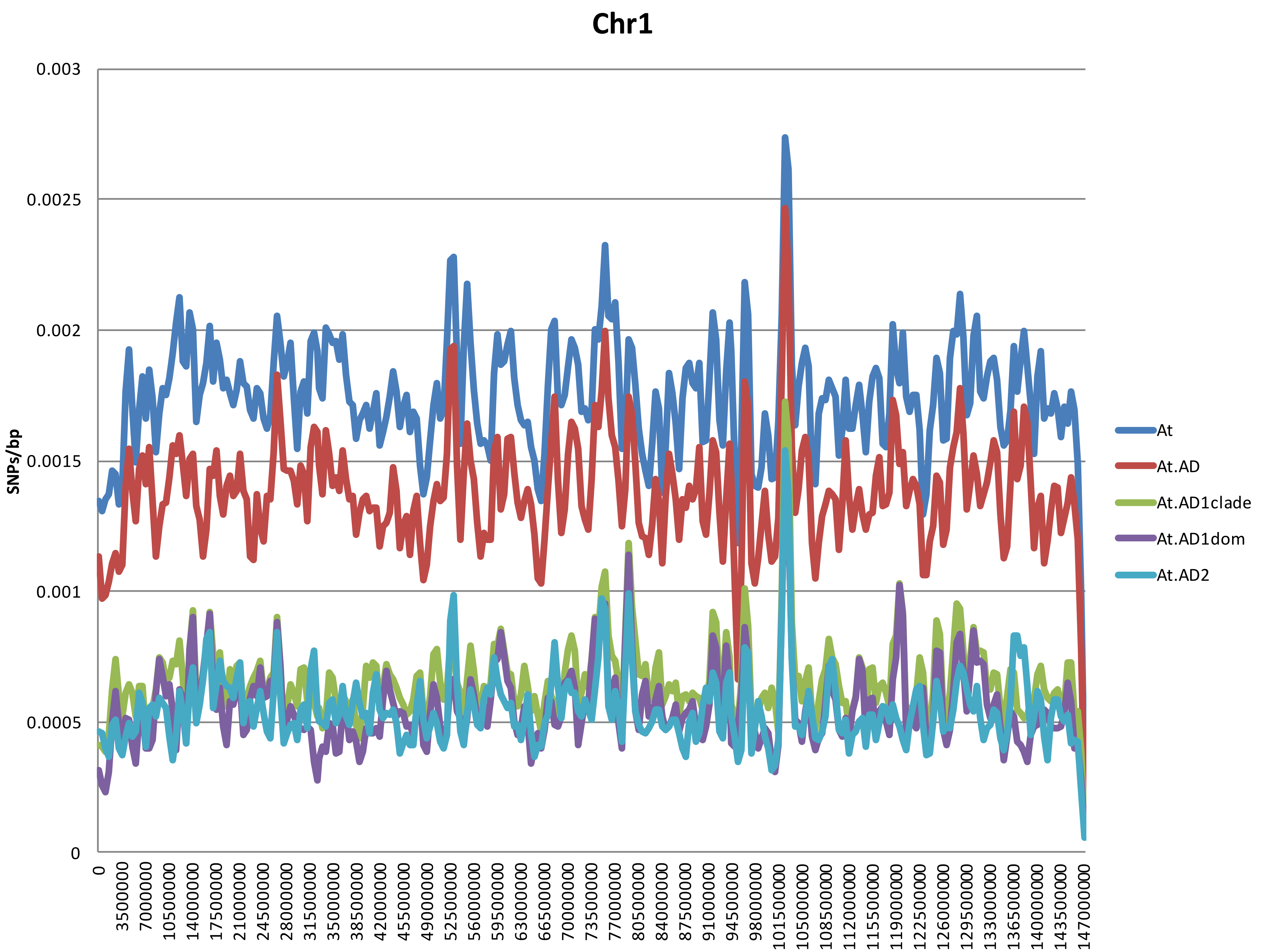

Chr1
0.003
0.0025
0.002
0.0015
0.001
0.0005
0
SNPs/bp
0
3500000
7000000
10500000
14000000
17500000
21000000
24500000
28000000
31500000
35000000
38500000
42000000
45500000
49000000
52500000
56000000
59500000
63000000
66500000
70000000
73500000
77000000
80500000
84000000
87500000
91000000
94500000
98000000
101500000
105000000
108500000
112000000
115500000
119000000
122500000
126000000
129500000
133000000
136500000
140000000
143500000
147000000
At
At.AD
At.AD1clade
At.AD1dom
At.AD2

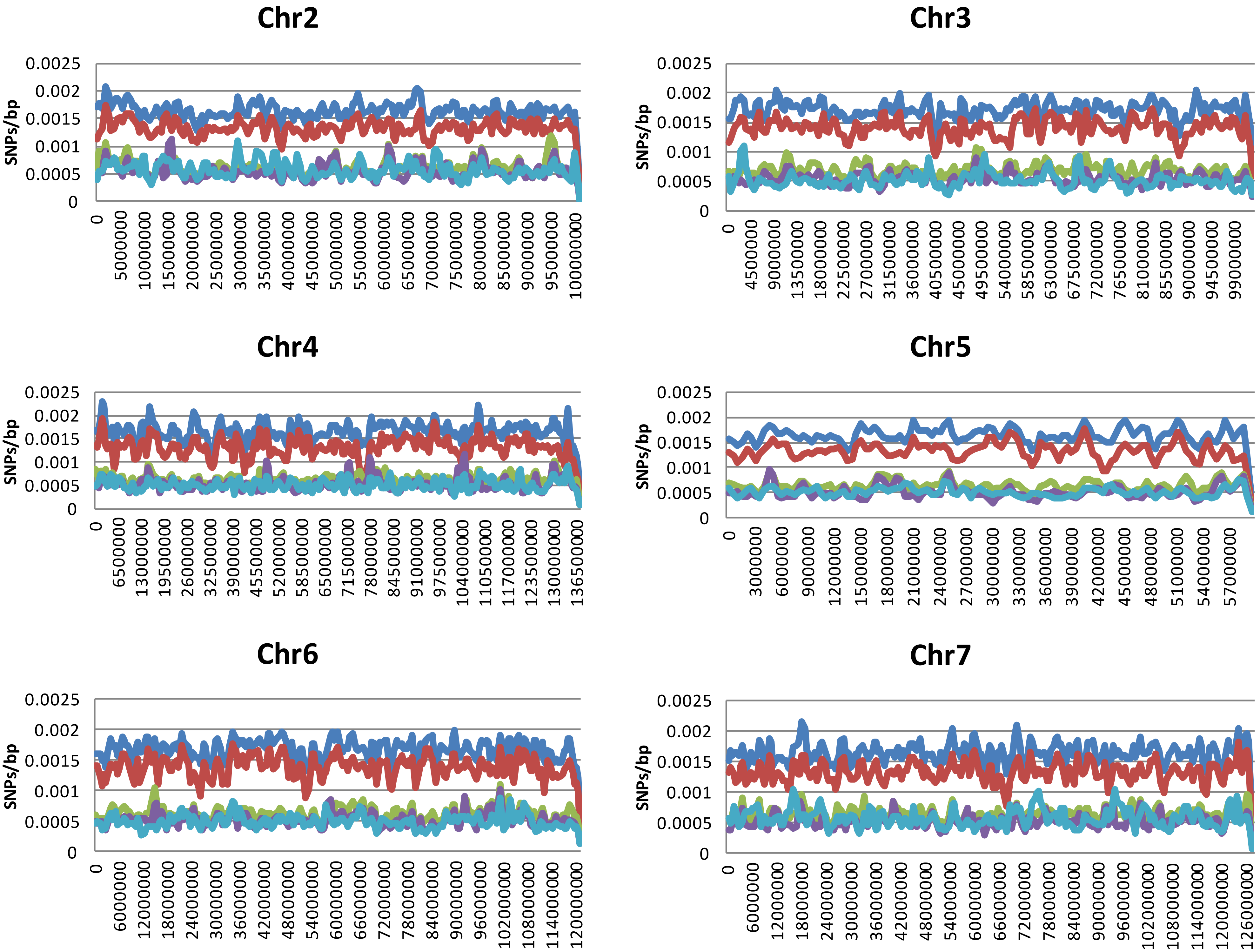

Chr2
SNPs/bp
0.0025
0.002
0.0015
0.001
0.0005
0
0
5000000
10000000
15000000
20000000
25000000
30000000
35000000
40000000
45000000
50000000
55000000
60000000
65000000
70000000
75000000
80000000
85000000
90000000
95000000
100000000
Chr3
SNPs/bp
0.0025
0.002
0.0015
0.001
0.0005
0
0
4500000
9000000
13500000
18000000
22500000
27000000
31500000
36000000
40500000
45000000
49500000
54000000
58500000
63000000
67500000
72000000
76500000
81000000
85500000
90000000
94500000
99000000
Chr4
SNPs/bp
0.0025
0.002
0.0015
0.001
0.0005
0
0
6500000
13000000
19500000
26000000
32500000
39000000
45500000
52000000
58500000
65000000
71500000
78000000
84500000
91000000
97500000
104000000
110500000
117000000
123500000
130000000
136500000
Chr5
SNPs/bp
0.0025
0.002
0.0015
0.001
0.0005
0
0
3000000
6000000
9000000
12000000
15000000
18000000
21000000
24000000
27000000
30000000
33000000
36000000
39000000
42000000
45000000
48000000
51000000
54000000
57000000
Chr6
SNPs/bp
0.0025
0.002
0.0015
0.001
0.0005
0
0
6000000
12000000
18000000
24000000
30000000
36000000
42000000
48000000
54000000
60000000
66000000
72000000
78000000
84000000
90000000
96000000
102000000
108000000
114000000
120000000
Chr7
SNPs/bp
0.0025
0.002
0.0015
0.001
0.0005
0
0
6000000
12000000
18000000
24000000
30000000
36000000
42000000
48000000
54000000
60000000
66000000
72000000
78000000
84000000
90000000
96000000
102000000
108000000
114000000
120000000
126000000

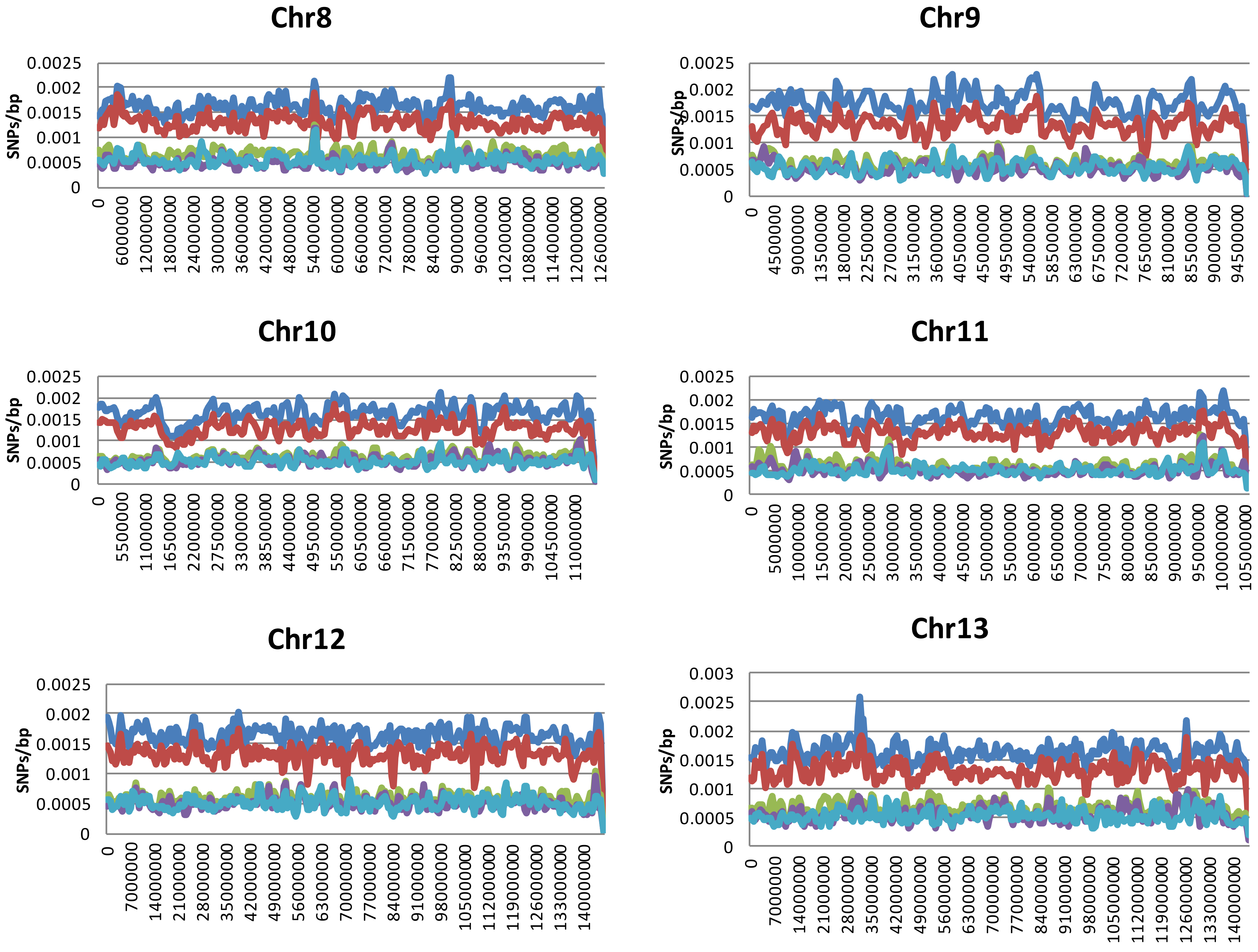
Chr8
SNPs/bp
0.0025
0.002
0.0015
0.001
0.0005
0
0 6000000 12000000 18000000 24000000 30000000 36000000 42000000 48000000 54000000 60000000 66000000 72000000 78000000 84000000 90000000 96000000 102000000 108000000 114000000 120000000 126000000
Chr9
SNPs/bp
0.0025
0.002
0.0015
0.001
0.0005
0
0 4500000 9000000 13500000 18000000 22500000 27000000 31500000 36000000 40500000 45000000 49500000 54000000 58500000 63000000 67500000 72000000 76500000 81000000 85500000 90000000 94500000
Chr10
SNPs/bp
0.0025
0.002
0.0015
0.001
0.0005
0
0 5500000 11000000 16500000 22000000 27500000 33000000 38500000 44000000 49500000 55000000 60500000 66000000 71500000 77000000 82500000 88000000 93500000 99000000 104500000 110000000
Chr11
SNPs/bp
0.0025
0.002
0.0015
0.001
0.0005
0
0 5000000 10000000 15000000 20000000 25000000 30000000 35000000 40000000 45000000 50000000 55000000 60000000 65000000 70000000 75000000 80000000 85000000 90000000 95000000 100000000 105000000
Chr12
SNPs/bp
0.0025
0.002
0.0015
0.001
0.0005
0
0 7000000 14000000 21000000 28000000 35000000 42000000 49000000 56000000 63000000 70000000 77000000 84000000 91000000 98000000 105000000 112000000 119000000 126000000 133000000 140000000
Chr13
SNPs/bp
0.003
0.0025
0.002
0.0015
0.001
0.0005
0
0 7000000 14000000 21000000 28000000 35000000 42000000 49000000 56000000 63000000 70000000 77000000 84000000 91000000 98000000 105000000 112000000 119000000 126000000 133000000 140000000

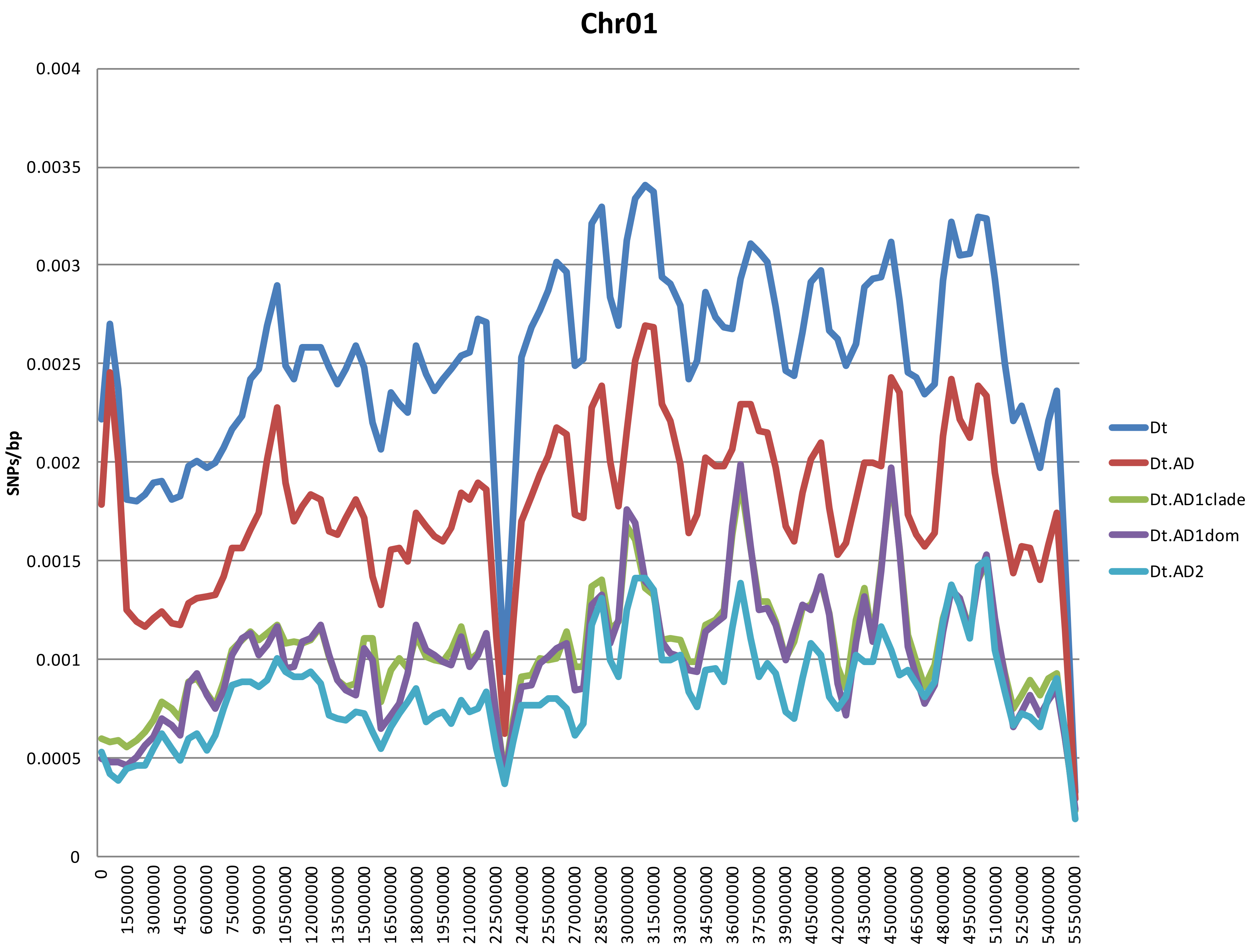

Chr01
SNPs/bp
0.004
0.0035
0.003
0.0025
0.002
0.0015
0.001
0.0005
0
0
1500000
3000000
4500000
6000000
7500000
9000000
10500000
12000000
13500000
15000000
16500000
18000000
19500000
21000000
22500000
24000000
25500000
27000000
28500000
30000000
31500000
33000000
34500000
36000000
37500000
39000000
40500000
42000000
43500000
45000000
46500000
48000000
49500000
51000000
52500000
54000000
55500000
Dt
Dt.AD
Dt.AD1clade
Dt.AD1dom
Dt.AD2

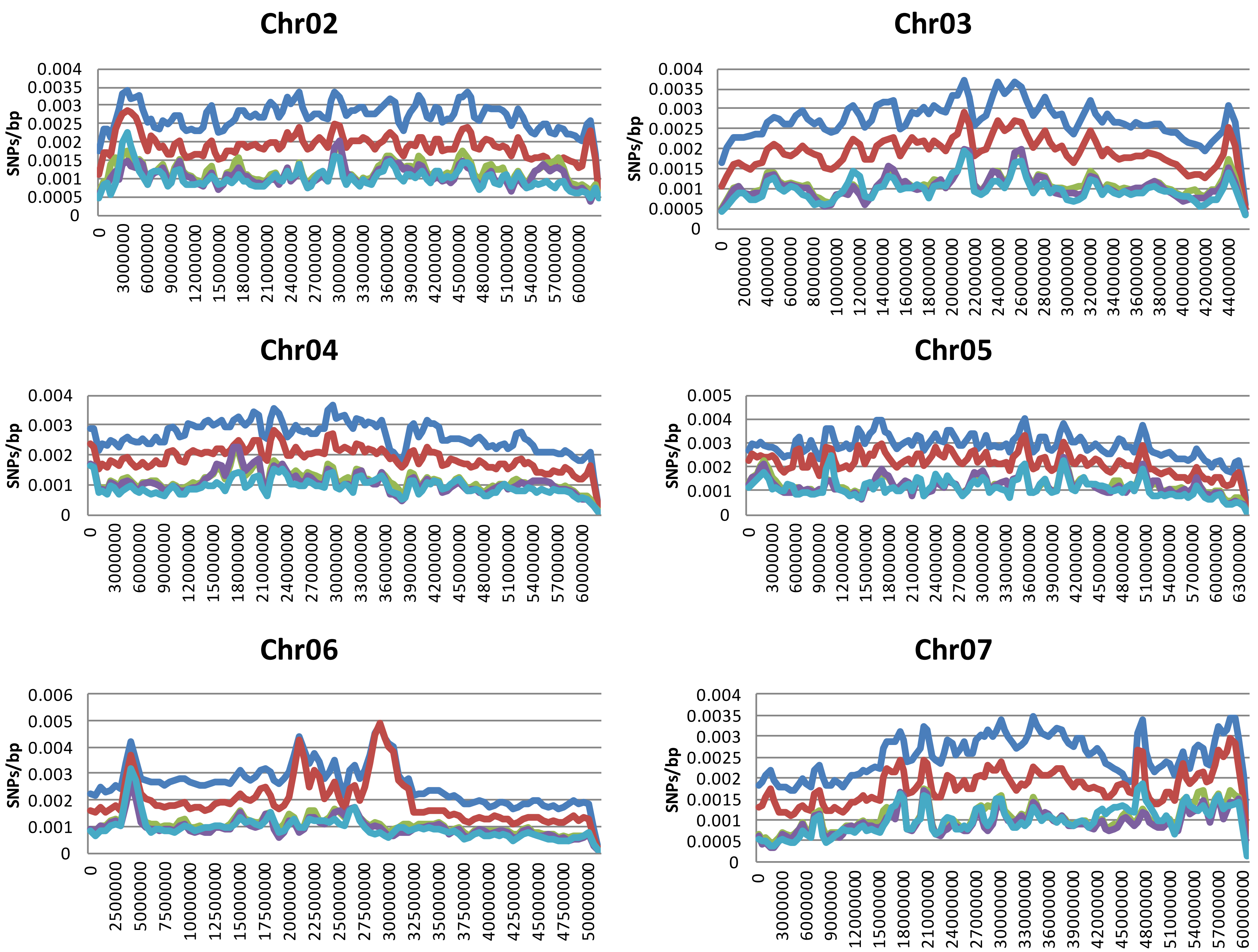
Chr02
SNPs/bp
0.004
0.0035
0.003
0.0025
0.002
0.0015
0.001
0.0005
0
0
3000000
6000000
9000000
12000000
15000000
18000000
21000000
24000000
27000000
30000000
33000000
36000000
39000000
42000000
45000000
48000000
51000000
54000000
57000000
60000000
Chr03
SNPs/bp
0.004
0.0035
0.003
0.0025
0.002
0.0015
0.001
0.0005
0
0
2000000
4000000
6000000
8000000
10000000
12000000
14000000
16000000
18000000
20000000
22000000
24000000
26000000
28000000
30000000
32000000
34000000
36000000
38000000
40000000
42000000
44000000
Chr04
SNPs/bp
0.004
0.003
0.002
0.001
0
0
3000000
6000000
9000000
12000000
15000000
18000000
21000000
24000000
27000000
30000000
33000000
36000000
39000000
42000000
45000000
48000000
51000000
54000000
57000000
60000000
Chr05
SNPs/bp
0.005
0.004
0.003
0.002
0.001
0
0
3000000
6000000
9000000
12000000
15000000
18000000
21000000
24000000
27000000
30000000
33000000
36000000
39000000
42000000
45000000
48000000
51000000
54000000
57000000
60000000
63000000
Chr06
SNPs/bp
0.006
0.005
0.004
0.003
0.002
0.001
0
0
2500000
5000000
7500000
10000000
12500000
15000000
17500000
20000000
22500000
25000000
27500000
30000000
32500000
35000000
37500000
40000000
42500000
45000000
47500000
50000000
Chr07
SNPs/bp
0.004
0.0035
0.003
0.0025
0.002
0.0015
0.001
0.0005
0
0
3000000
6000000
9000000
12000000
15000000
18000000
21000000
24000000
27000000
30000000
33000000
36000000
39000000
42000000
45000000
48000000
51000000
54000000
57000000
60000000

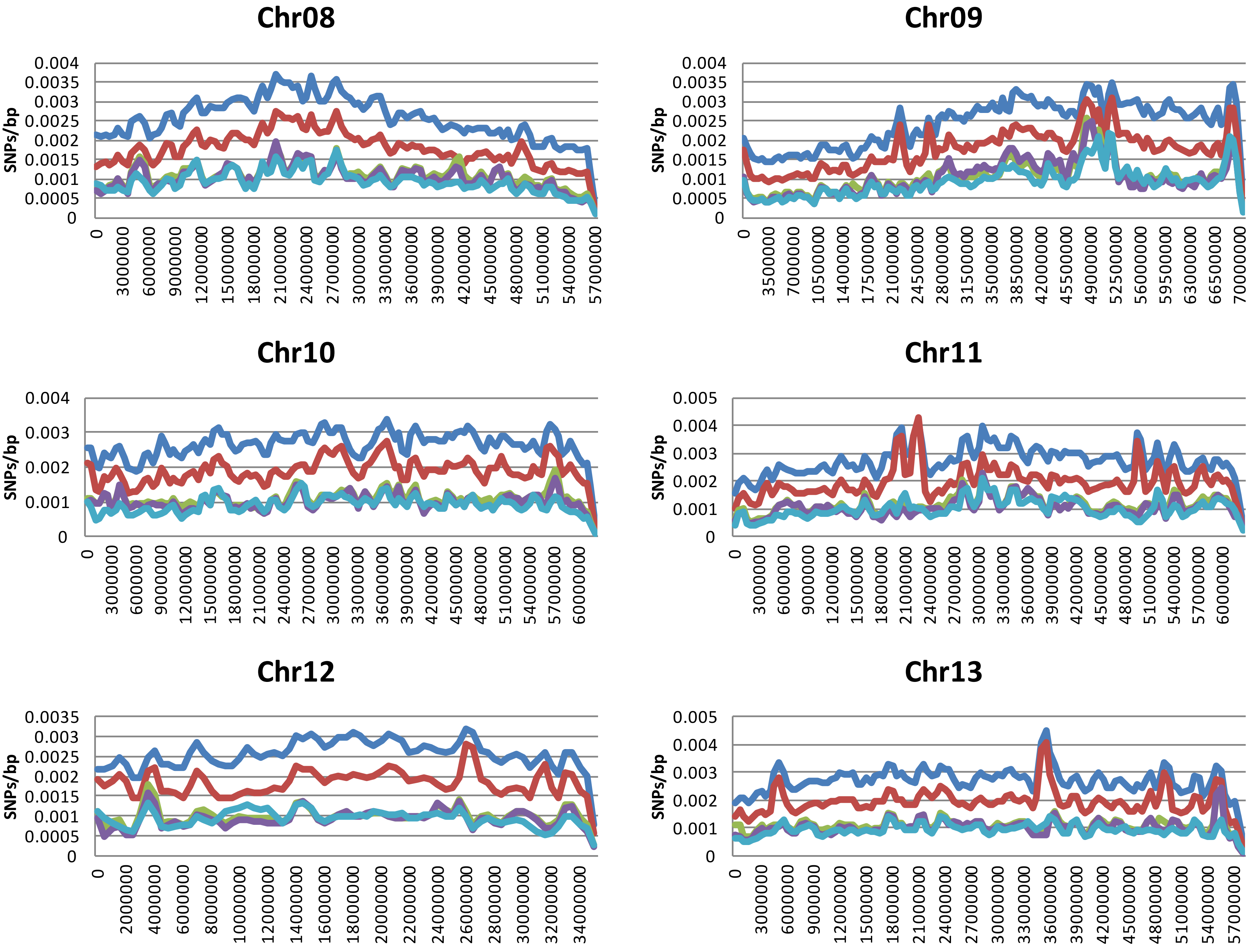

Chr08
SNPs/bp
0.004
0.0035
0.003
0.0025
0.002
0.0015
0.001
0.0005
0
0
3000000
6000000
9000000
12000000
15000000
18000000
21000000
24000000
27000000
30000000
33000000
36000000
39000000
42000000
45000000
48000000
51000000
54000000
57000000
Chr09
SNPs/bp
0.004
0.0035
0.003
0.0025
0.002
0.0015
0.001
0.0005
0
0
3500000
7000000
10500000
14000000
17500000
21000000
24500000
28000000
31500000
35000000
38500000
42000000
45500000
49000000
52500000
56000000
59500000
63000000
66500000
70000000
Chr10
SNPs/bp
0.004
0.003
0.002
0.001
0
0
3000000
6000000
9000000
12000000
15000000
18000000
21000000
24000000
27000000
30000000
33000000
36000000
39000000
42000000
45000000
48000000
51000000
54000000
57000000
60000000
Chr11
SNPs/bp
0.005
0.004
0.003
0.002
0.001
0
0
3000000
6000000
9000000
12000000
15000000
18000000
21000000
24000000
27000000
30000000
33000000
36000000
39000000
42000000
45000000
48000000
51000000
54000000
57000000
60000000
Chr12
SNPs/bp
0.0035
0.003
0.0025
0.002
0.0015
0.001
0.0005
0
0
2000000
4000000
6000000
8000000
10000000
12000000
14000000
16000000
18000000
20000000
22000000
24000000
26000000
28000000
30000000
32000000
34000000
Chr13
SNPs/bp
0.005
0.004
0.003
0.002
0.001
0
0
3000000
6000000
9000000
12000000
15000000
18000000
21000000
24000000
27000000
30000000
33000000
36000000
39000000
42000000
45000000
48000000
51000000
54000000
57000000
