# Supplementary figures and images for: DNA Sequence Evolution and Rare Homoeologous Conversion in Tetraploid Cotton
Source: PLoS Genet. 2016 May 11;12(5):e1006012. doi: 10.1371/journal.pgen.1006012 (PMC4864293; doi:10.1371/journal.pgen.1006012)

S2 Fig

A

Length

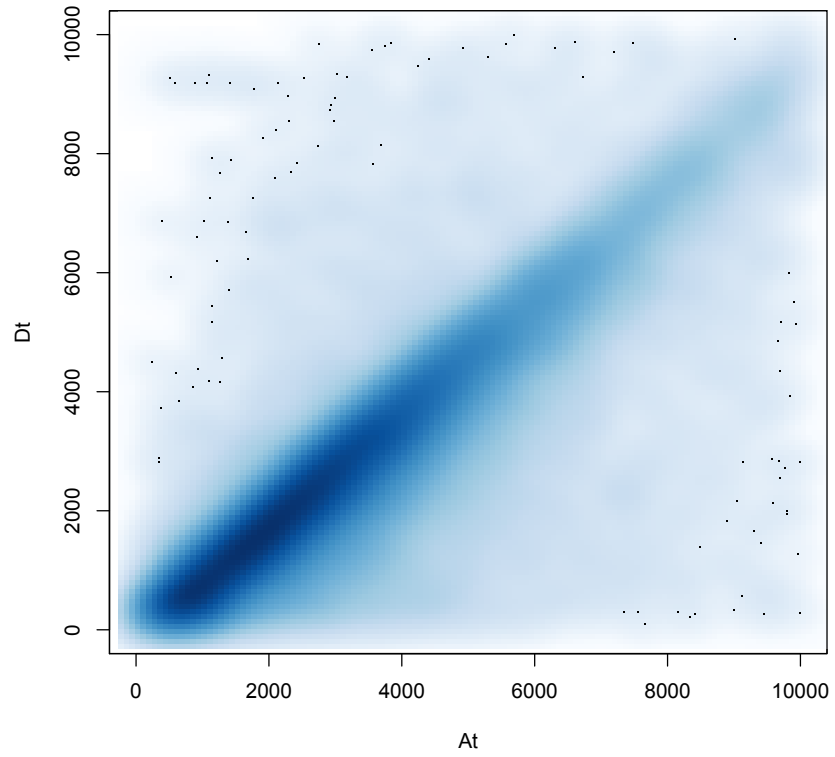

B

Density

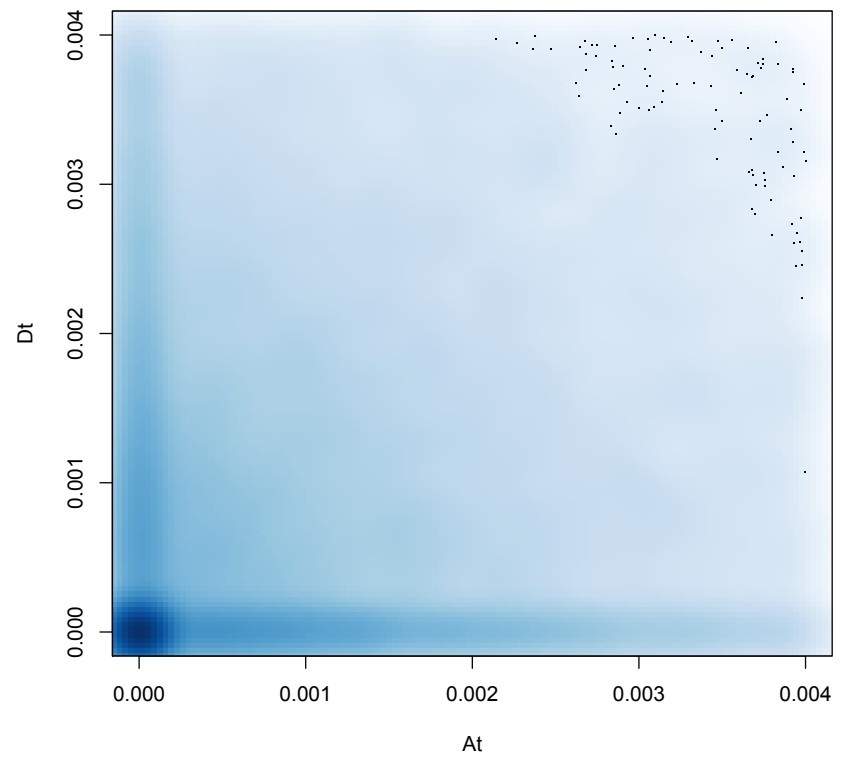

Supplement: S2 Fig — The density of allele-SNPs was weakly correlated among allotetraploids (Pearson r2 = 0.321, p-value < 2.2e-16; Supp. Fig 2A) and among AD1 cultivars (Pearson r2 = 0.261, p-value < 2.2e-16; Supp. Fig 2B). (PDF) [file pgen.1006012.s008.pdf]

S3 Fig

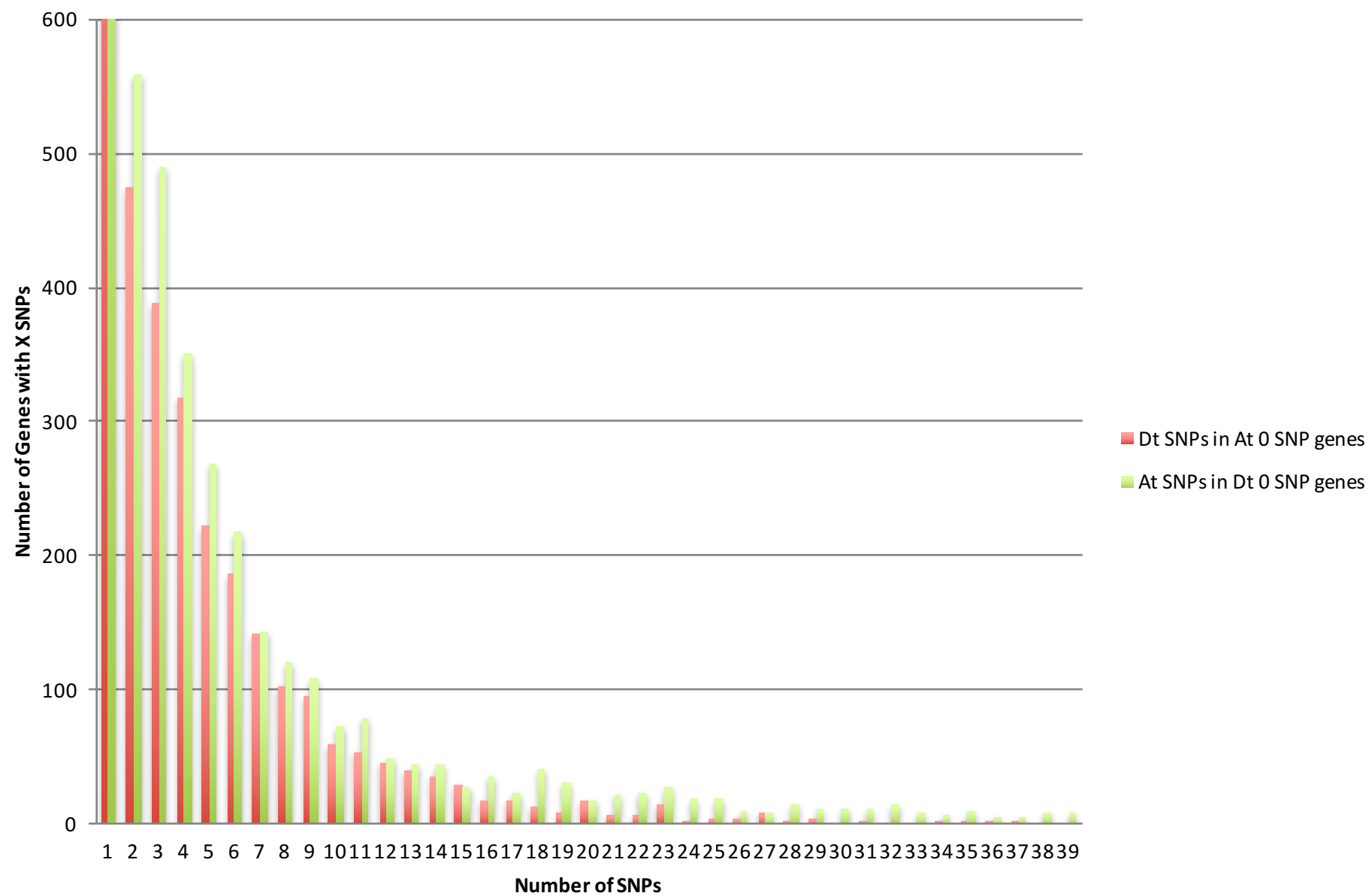

Supplement: S3 Fig — The number of SNPs in one gene when its homoeolog has 0 SNPs. Red is for genes that have 0 SNPs in the AT-genome homoeolog; green is for genes that have 0 SNPs in the DT-genome homoeolog. To identify homoeolog pairs in the annotations of the A2 and D5 reference sequences, we used BLASTP with a maximum e-value of 10−20 to compare the peptide sequences of annotated A2 and D5 genes [43]. (PDF) [file pgen.1006012.s009.pdf]
